# Supplementary material for: Study protocol for an exploratory interventional study investigating the feasibility of video-based non-contact physiological monitoring in healthy volunteers by Mapping Of Lower Limb skIn pErfusion (MOLLIE)
Source: BMJ Open. 2020 Jun 11;10(6):e036235. doi: 10.1136/bmjopen-2019-036235 (PMC7295406; doi:10.1136/bmjopen-2019-036235)
Supplement: Supplementary data [file bmjopen-2019-036235supp001.pdf]

Critical Care Research Group (Kadoorie Centre)
Level 3, John Radcliffe Hospital, Oxford
Nuffield Department of Clinical Neurosciences
Tel: +44 (0)1865 231449
Email: mirae.harford@ndcn.ox.ac.uk

PARTICIPANT INFORMATION SHEET

Mapping Of Lower Limb skIn pErfusion (MOLLIE)

Ethics Approval Reference: R63796/RE001
Version 2.0
Date: May 2019

We'd like to invite you to take part in our research study. Before you decide, it is important that you understand why the research is being done and what it would involve for you. Please take time to read this information, and discuss it with others if you wish. If there is anything that is not clear, or if you *would like more information, please ask us.*

What is the purpose of the study?

We are trialling a new approach to monitoring how well a patient's heart and their cardiovascular system is functioning. Many established methods of looking at this use invasive monitors or semi-invasive monitors. Non-invasive monitors are less uncomfortable for patients but still require direct contact with the patients with risks of infection and skin irritation. The new method uses video cameras that can detect the way blood arrives at the skin surface, which is not visible to the naked eye. We believe that using this method we may be able to develop a way we can monitor patients without any invasive lines or contacts to their skin.

In order to explore this further, we are aiming to study the way skin is perfused in health, followed by simulating some of the changes that may occur when patients are less well. We are particularly interested in leg skin perfusion and that is the focus of this study. We are planning on using two methods to study this:

- Increasing and decreasing the amount of blood delivered to your skin surface by changing the diameter of blood vessels close to the skin surface. This will be done by using two drugs called phenylephrine and glyceryl trinitrate (GTN). Both of these drugs have been used safely in healthy volunteers and they are frequently used in hospitals. After a recovery period, we will roll a tight-fitting doughnut-shaped ring over the leg. It will feel like wearing tight-fitting socks or tights temporarily and should not be uncomfortable. The ring will act as a

tourniquet once placed around the thigh. After one minute this tourniquet will be released and the re-filling of these vessels will be monitored using the video camera.

Why have I been invited?

You have been invited because you are a healthy adult, aged between 18 and 65, do not have any known pre-existing heart problems, not on any regular medication (except oral contraception), and have expressed interest in taking part in the study.

You will not be able to take part if:

- there are any reasons why you cannot use the monitoring equipment (camera monitor or monitors which are attached to your body via sticky adhesive pads) due to skin disorders such as eczema, scleroderma or psoriasis
- you have had any degree of leg amputation
- you have had any previous surgery to the thigh/knee/lower leg (except knee arthroscopy)
- you are allergic to silver chloride ECG pads
- you have hyperthyroidism
- you are pregnant or breastfeeding
- you have neurological conditions affecting blood flow to the leg
- you have known heart conditions
- you suffer from severe headaches

Do I have to take part?

No. It is up to you to decide if you want to take part in the study. We will describe the study and go through this information sheet with you to answer any questions you may have.

If you agree to take part, we will ask you to sign a consent form and give you a copy for you to keep.

If you change your mind, you are free to withdraw from the study at any time, without having to provide a reason.

What will happen to me if I decide to take part?

The study will take part in the Cardiovascular Clinical Research Facility (John Radcliffe Hospital, Oxford) and will involve one single visit, unless we are unable to collect data needed on this visit or you ask us to discontinue the study and reschedule for another day. We expect one full session to take approximately 2.5 hours.

As we know that the video monitors are very sensitive to body hair, we will ask you to remove hair on your leg for the study. As shaving or waxing during the study visit may temporarily increase blood flow to the skin surface, we will ask you to do this at least 24 hours before the study visit. If you have hair removal equipment at home,

you can use your usual method. If not, we will discuss this with you before the study visit and agree on a plan. If required, we can supply body hair removal equipment (for shaving or waxing) that you can use.

Prior to starting, we will go through this information with you again and ask if you have any questions. Following that we will ask you some brief medical questions to ensure you are fit and well and examine your heart, lungs, and abdomen to make sure you are fit to take part in the study. Once we are happy that you can partake in the study, we will take some basic measurements from you. This will include your height, weight, and your skin colour from your arm according to a skin colour chart. This is so that the performance of the camera monitor can be compared to your skin tone. As we are recording from your legs, we will ask you to wear shorts so that the skin over your thigh is visible.

We will ask you to lie down on the bed and place a small drip on your arm. This is called a cannula and involves a small needle to ensure it sits inside your vein. The needle is removed after the drip is placed and a small plastic tube will then remain in your arm. The purpose of a cannula is to allow controlled infusion of the drugs to change the diameter of your blood vessels in a safe way. The cannulation will be completed by a trained doctor using sterile equipment. Once inserted, the cannula placement will be checked by flushing up to 10mls of normal saline (sterile fluid with small amount of salt to match the salt levels in the blood) and it will be secured using a specially designed plaster.

The cannula will then be connected to a flexible tube which will be connected to the drug infusion pump. At this point, the infusion will not be running.

The study will involve multiple methods of monitoring the heart and the circulation as described below:

- Video monitors: this is just like a normal video camera and will be mounted onto a trolley next to the examination bed where you will be lying. The camera will be set up after you are in the room and you will be warned when the recording is about to start. The camera is only recording your legs; your face and upper body will not be recorded. While the camera is recording we will ask you to maintain the same position as much as possible, as the monitor is very sensitive to movement. The camera will continue to record throughout the entire study visit and will be analysed at a later point.
- Thoracic bioimpedance monitor: This looks at the way your heart is pumping the blood around the body by measuring the total amount of water inside the chest cavity. The machine does this by passing a tiny amount of electrical signal between the leads which will be placed as sticky adhesives on either side of your neck and your lower chest. The amount of electrical signal passed is very small and you will not be aware of it. This is a continuous monitor and will continue to record throughout the study period.

Harford M, *et al. BMJ Open* 2020; 10:e036235. doi: 10.1136/bmjopen-2019-036235

- A non-invasive blood pressure monitor: This is a cuff that will be placed around your right arm which will intermittently inflate to measure your blood pressure.
- An oxygen saturation probe: This is an earlobe probe that will be placed on your left earlobe. The probe measures the amount of oxygen your blood is carrying using red and infrared light.
- Stowood Blackshadow monitor: This is a device which measures your breathing rate via two belts across your chest and a monitor placed just under your nostrils, and your heart rate and oxygen saturations using sticky adhesives on your chest and a finger probe.

Once all equipment is attached and we are ready to record, we will let you know when the study will start. For the first ten minutes, we will ask you to lie down as still as possible while we take some background measurements. We know from previous studies that talking during this period can cause fine movements in the body which reduces the quality of the images. Therefore, we will ask you to maintain silence during this period.

After ten minutes, we will administer one of the drugs. Each will last for 15 minutes and there will be a break of 20 minutes before the next drug is given. While the drug is being given, your blood pressure will be measured every minute. The drug infusion rate will slowly increase using our protocol, and we will stop increasing the rate once your blood pressure changes by 30% from baseline.

Once the drug infusions are complete and you have had a further break of 20 minutes, we will apply a tourniquet to one of your legs after passing a wrap balloon over your leg to empty out the blood vessels near the surface of your skin. After the tourniquet is applied for approximately 30 seconds, we will let the cuff down while taking a video of your leg.

This is the end of the study and after safe removal of the cannula, we will observe you for a further five minutes. We will offer you some refreshments during this observation period. If you are feeling well, you will be free to go.

What drugs will I be given?

- Phenylephrine is a drug which has two effects; it can increase your blood pressure and lower your heart rate. It raises blood pressure by reducing the diameter of small blood vessels of arms and legs). The infusion is frequently used in women having caesarean sections under spinal anaesthetic to keep the blood pressure stable, and has been used at higher doses than planned in this study in other healthy volunteer studies.
- Glyceryl trinitrate (GTN) is a drug which has the opposite effect to that of phenylephrine. It reduces blood pressure by dilating the blood vessels. It is used in clinical settings to reduce blood pressure and to reduce the work of

the heart when people have heart problems, and has been used in healthy volunteer studies at higher doses than planned in this study. Its main side effect is that in some people it can cause a headache. If this occurs, let us know and we will not further increase the dose.

Both of these drugs are short acting and will wash out of your system quickly once the infusions stop. It is very unlikely that you will have ongoing effects from the drug after leaving the study visit.

What should I consider?

You may participate in other research studies while you are taking part in this study.

Are there any possible disadvantages or risks from taking part?

The monitoring methods described are very safe and non-invasive. They have been trialled in healthy people and in patients without any problems.

The cannulation procedure and its presence may feel uncomfortable. We will not keep the cannula in for any longer than necessary. Once removed, the cannula may leave a small bruise but will heal quickly within 2-3 days.

There is a possibility that you may experience side effects from the drug infusions. The side effects occur because of the transient changes in blood pressure. More specifically, the most common side effects of the two drugs are headaches, flushing, and nausea. These effects should disappear once the drugs washout from your system. Approximately 95% of the drugs will be out of your system by the time you leave the study visit and the remainder (which is not enough to cause you any symptoms or harm) will leave your body over a few hours following the study visit. Other risks associated with very high or low blood pressure will be minimised by monitoring your blood pressure closely at one minute intervals and making small step increases in drug doses being given. We will not allow your blood pressure to deviate more than 30% from baseline which falls within ranges expected during activities such as exercise and sleep.

The tourniquet placed on the thigh will be inflated to high pressure to stop blood flow. This can be uncomfortable but will only last for 30 seconds before the cuff is deflated.

What are the possible benefits of taking part?

There is no direct benefit for your participation in this study. We hope that the results from this research will help us develop a new method of monitoring critically unwell patients in the hospital in a more comfortable way.

Harford M, *et al. BMJ Open* 2020; 10:e036235. doi: 10.1136/bmjopen-2019-036235

Will my taking part in the study be kept confidential?

Yes. All study data will be entered on a spreadsheet and you will only be identifiable by a unique study specific number and/or code in any database. The name and any other identifying detail about you will not be included in any study data electronic file.

The raw video data of your legs will be stored in a format that is only readable by the review software and will be recorded onto a storage device that will be physically secured at Kadoorie Centre (behind two separate locked doors with restricted access) and the Institute of Biomedical Engineering (behind a locked door with restricted access and 24 hour CCTV monitoring). Access to this storage device will be limited to our research team. Video data analysis will be completed by our collaborators at the Oxford University Centre of Excellence for Medical Engineering who have designed the equipment being used for the study. They are based in the Institute of Biomedical Engineering.

Will I be reimbursed for taking part?

Should you incur any expenses as a result of the study, we will reimburse you as is reasonable (e.g. travel expenses). Additionally, once you complete the study we will give you a £50 voucher.

What will happen to my data?

The information you provide as part of the study is the **research data**. Any research data from which you can be identified (e.g. your name, date of birth), is known as **personal data**. This includes more sensitive categories of personal data (**special category data**) such as your racial or ethnic origin or data concerning your health. This does not include data where the identity has been removed (anonymous data).

We will minimise our use of personal and sensitive data in the study as much as possible.

Consent forms (which include your name) will be stored in the Kadoorie Centre for Critical Care Research and Education within the John Radcliffe Hospital, behind two restricted access doors. All other research data will be stored in the Kadoorie Centre or in the Institute of Biomedical Engineering in the Department of Engineering Science, University of Oxford on a secure local server. Access to this server is restricted to the members of our study team only and the Institute of Biomedical Engineering is accessed via two restricted access doors. Research data will be anonymised. Personal/sensitive data will be stored in the Kadoorie Centre.

The researcher and the research team will have access to personal/sensitive/research data.

We would like your permission to use anonymised images or videos of your legs in research publications. While it is not possible to fully anonymise images of legs, we will not use images with any distinguishing features (e.g. distinctive scar) which may make you identifiable from the image.

All research data and records will be stored for a minimum of 5 years after publication or public release of the work of the research.

We would like your permission to use anonymised data in future studies, and to share data with other researchers (e.g. online database) both inside and outside the European Union. All personal information that could identify you will be removed or changed before information is shared with other researchers or results are made public.

The University of Oxford is the data controller with respect to your personal data and, as such, will determine how your personal data is used in the study. The University will process your personal data for the purpose of the research outlined above. Research is a task that we perform in the public interest.

For further information about your rights with respect to your personal data is available http://www.admin.ox.ac.uk/councilsec/compliance/gdpr/individualrights/.

What will happen if I don't want to carry on with the study?

Participation is voluntary you may change your mind at any stage. If you withdraw from the study, unless you state otherwise, any non-identifiable video data which have been collected whilst you have been in the study will be used for research as detailed in this participant information sheet. You are free to request that your video samples are destroyed at any time during or after the study.

What will happen to the results of this study?

It will not be possible to identify you from any report or publication placed in the public domain.

Once the study is complete including the analysis of results, the data will be used for publication in scientific journals and may be presented at conferences. It is also predicted that the current study will be used as basis for future similar studies.

Some of the research being undertaken will also contribute to the fulfilment of an educational requirement (i.e. a doctoral thesis).

What if we find something unexpected?

It is important to note that the examination and monitoring in the study are carried out for research purposes rather than for diagnosis. Therefore, participating in the study is not a substitute for a doctor's appointment. Occasionally, however, a possible abnormality may be detected. In this case, we will inform you of the findings and inform the lead investigator (Professor Peter Watkinson). If the lead investigator felt that the abnormality was medically important, you will be recommended to see your general practitioner. All information about you is kept strictly confidential.

There is no need to inform your General Practitioner/family doctor of your participation in the study unless you would like to. In the unlikely event that any findings need urgent treatment or investigation, we will inform you of this and may speak to your GP with your permission.

What if there is a problem?

If a participant in research is ever considered to have suffered harm through their participation, the University has arrangements in place to provide for compensation. If you have a concern about any aspect of this study, please speak to the relevant researcher (01865 231449) or their supervisor (01865 572609), who will do their best to answer your query. The researcher should acknowledge your concern within 10 working days and give you an indication of how they intend to deal with it. If you remain unhappy or wish to make a formal complaint, please contact the Chair of the Medical Sciences Interdivisional Research Ethics Committee (MS IDREC) at the University of Oxford who will seek to resolve the matter in a reasonably expeditious manner: Email: ethics@medsci.ox.ac.uk; Address: Research Services, University of Oxford, Wellington Square, Oxford OX1 2JD.

Who is organising and funding the study?

This study is being organised by a group of researchers specialising in critical care research at the Nuffield Department of Clinical Neuroscience, based at Kadoorie Centre in John Radcliffe Hospital.

The study is funded by the Oxford Biomedical Research Centre, which is a part of National Institute for Health Research (NIHR).

Who has reviewed the study?

This study has been reviewed by, and received ethics clearance through, the University of Oxford Central University Research Ethics Committee (Reference number: R63796/RE001).

Participation in future research:

We will not keep your contact details to contact you regarding future research. If you would like to know more about participating in future research projects, please contact Dr Mirae Harford.

Further information and contact details:

Please contact Dr Mirae Harford by email: mirae.harford@ndcn.ox.ac.uk.

Thank you for reading this information and for considering taking part.

Harford M, *et al. BMJ Open* 2020; 10:e036235. doi: 10.1136/bmjopen-2019-036235
