# Supplementary material for: Study protocol for an exploratory interventional study investigating the feasibility of video-based non-contact physiological monitoring in healthy volunteers by Mapping Of Lower Limb skIn pErfusion (MOLLIE)
Source: BMJ Open. 2020 Jun 11;10(6):e036235. doi: 10.1136/bmjopen-2019-036235 (PMC7295406; doi:10.1136/bmjopen-2019-036235)
Supplement: Supplementary data [file bmjopen-2019-036235supp002.pdf]

Critical Care Research Group
Nuffield Department of Clinical Neurosciences
University of Oxford

Lead investigator: Professor Peter Watkinson
Peter.watkinson@ndcn.ox.ac.uk
Primary researcher: Mirae Harford
Oxford telephone number: 01865 231449
Oxford e-mail: mirae.harford@ndcn.ox.ac.uk

PARTICIPANT CONSENT FORM

CUREC Approval Reference: R63796/RE001

Mapping Of Lower Limb skIn pErfusion (MOLLIE)

Purpose of Study: To investigate leg skin perfusion at rest and changes created to the perfusion pattern in response to phenylephrine and glyceryl trinitrate (GTN) infusions.

		Please initial each box
1	I confirm that I have read and understand the information sheet version 2.0 dated May 2019 for the above study. I have had the opportunity to consider the information carefully, ask questions and have had these questions answered satisfactorily.	☐
2	I understand that my participation is voluntary and that I am free to withdraw at any time, without giving any reason, and without any adverse consequences or academic penalty.	☐
3	I have been advised about the potential risks associated with taking part in this research and have taken these into consideration before consenting to participate. I understand that the study visit involves video equipment and the recordings will be saved for subsequent analysis.	☐
4	I have been advised as to what I need to do for this research (especially with regard to the phenylephrine and GTN administration) and I agree to follow the instructions given to me. I understand that taking part will involve having a drip and application of a tourniquet on one leg which will stay inflated for one minute.	☐
5	To the best of my knowledge, I do not meet any of the exclusion criteria outlined in the information sheet for this research. If this changes at a later date during study participation, I agree to notify the researchers immediately.	☐
6	I understand that data collected during the study may be looked at by designated individuals from the University of Oxford. I give permission for these individuals to access my data.	☐
8	I understand who will have access to personal data provided, how the data will be stored and what will happen to the data at the end of the project.	☐
9	I agree for data collected in this study to be shared with other researchers, including those working outside of the EU, to be used in other research studies. I understand that any data shared will be fully anonymised so that I cannot be identified.	☐

Harford M, *et al. BMJ Open* 2020; 10:e036235. doi: 10.1136/bmjopen-2019-036235

10	I understand how this research will be written up and published. I agree to the use of anonymised data (including videos) in research publications.	☐
11	I understand that this project has been reviewed by, and received ethics clearance through, the University of Oxford Central University Research Ethics Committee.	☐
12	I understand how to raise a concern or make a complaint.	☐
13	I agree to take part in the study	☐

________________	dd / mm / yyyy	________________
Name of Participant	Date	Signature

________________	dd / mm / yyyy	________________
Name of person taking consent	Date	Signature

Harford M, *et al. BMJ Open* 2020; 10:e036235. doi: 10.1136/bmjopen-2019-036235
