# Supplementary material for: Study protocol for an exploratory interventional study investigating the feasibility of video-based non-contact physiological monitoring in healthy volunteers by Mapping Of Lower Limb skIn pErfusion (MOLLIE)
Source: BMJ Open. 2020 Jun 11;10(6):e036235. doi: 10.1136/bmjopen-2019-036235 (PMC7295406; doi:10.1136/bmjopen-2019-036235)
Supplement: Author's manuscript [file bmjopen-2019-036235.draft_revisions.pdf]

BMJ Open is committed to open peer review. As part of this commitment we make the peer review history of every article we publish publicly available.

When an article is published we post the peer reviewers' comments and the authors' responses online. We also post the versions of the paper that were used during peer review. These are the versions that the peer review comments apply to.

The versions of the paper that follow are the versions that were submitted during the peer review process. They are not the versions of record or the final published versions. They should not be cited or distributed as the published version of this manuscript.

BMJ Open is an open access journal and the full, final, typeset and author-corrected version of record of the manuscript is available on our site with no access controls, subscription charges or pay-per-view fees (http://bmjopen.bmj.com).

If you have any questions on BMJ Open's open peer review process please email info.bmjopen@bmj.com

BMJ Open

A study protocol for an exploratory interventional study investigating the feasibility of video-based non-contact physiological monitoring in healthy volunteers by Mapping Of Lower Limb skIn pErfusion (MOLLIE)

Journal:	*BMJ Open*
Manuscript ID	bmjopen-2019-036235
Article Type:	Protocol
Date Submitted by the Author:	06-Dec-2019

Complete List of Authors:	Harford, Mirae; University of Oxford, Nuffield Department of Clinical Neurosciences; University of Oxford, Institute of Biomedical Engineering, Department of Engineering Science<br>Areia, Carlos; University of Oxford, Nuffield Department of Clinical Neurosciences<br>Villarroel, Mauricio; University of Oxford, Institute of Biomedical Engineering, Department of Engineering Science<br>Jorge, Joao; University of Oxford, Institute of Biomedical Engineering, Department of Engineering Science<br>Finnegan, Eoin; University of Oxford, Institute of Biomedical Engineering, Department of Engineering Science<br>Davidson, Shaun; University of Oxford, Institute of Biomedical Engineering, Department of Engineering Science<br>Mahdi, Adam; University of Oxford, Institute of Biomedical Engineering, Department of Engineering Science<br>Young, Duncan; Oxford University, Nuffield Department of Clinical Neurosciences<br>Tarassenko, Lionel; University of Oxford, Institute of Biomedical Engineering, Department of Engineering Science<br>Watkinson, Peter; Oxford University, Nuffield Department of Clinical Neurosciences

Keywords:	Anatomy < BASIC SCIENCES, CLINICAL PHYSIOLOGY, CLINICAL PHARMACOLOGY

SCHOLARONE™
Manuscripts

I, the Submitting Author has the right to grant and does grant on behalf of all authors of the Work (as defined in the below author licence), an exclusive licence and/or a non-exclusive licence for contributions from authors who are: i) UK Crown employees; ii) where BMJ has agreed a CC-BY licence shall apply, and/or iii) in accordance with the terms applicable for US Federal Government officers or employees acting as part of their official duties; on a worldwide, perpetual, irrevocable, royalty-free basis to BMJ Publishing Group Ltd ("BMJ") its licensees and where the relevant Journal is co-owned by BMJ to the co-owners of the Journal, to publish the Work in this journal and any other BMJ products and to exploit all rights, as set out in our licence.

The Submitting Author accepts and understands that any supply made under these terms is made by BMJ to the Submitting Author unless you are acting as an employee on behalf of your employer or a postgraduate student of an affiliated institution which is paying any applicable article publishing charge ("APC") for Open Access articles. Where the Submitting Author wishes to make the Work available on an Open Access basis (and intends to pay the relevant APC), the terms of reuse of such Open Access shall be governed by a Creative Commons licence – details of these licences and which Creative Commons licence will apply to this Work are set out in our licence referred to above.

Other than as permitted in any relevant BMJ Author's Self Archiving Policies, I confirm this Work has not been accepted for publication elsewhere, is not being considered for publication elsewhere and does not duplicate material already published. I confirm all authors consent to publication of this Work and authorise the granting of this licence.

For peer review only

A study protocol for an exploratory interventional study investigating the feasibility of video-based non-contact physiological monitoring in healthy volunteers by Mapping Of Lower Limb skIn pErfusion (MOLLIE)

Mirae Harford[1,2]

Carlos Areia[1]

Maurico Villarroel[2]

Joao Jorge[2]

Eoin Finnegan[2]

Shaun Davidson[2]

Adam Mahdi[1,2]

John Duncan Young[1]

Lionel Tarassenko[2]

Peter Watkinson[1]

1. Critical Care Research Group, Kadoorie Centre for Critical Care Research and Education, Nuffield Department of Clinical Neurosciences, University of Oxford, Oxford, United Kingdom
2. Institute of Biomedical Engineering, Department of Engineering Science, University of Oxford, Oxford, United Kingdom

Corresponding author: Mirae Harford (mirae.harford@ndcn.ox.ac.uk), 01865 332101

Word count: 3456

For peer review only

Abstract

Introduction: Skin perfusion varies in response to changes in the circulatory status. Blood flow to skin is reduced during haemodynamic collapse secondary to peripheral vasoconstriction, while increased skin perfusion is frequently observed when haemodynamics improve with resuscitation. These changes in perfusion may be monitored using non-contact image-based methods. Previous camera-derived physiological measurements have focused on accurate vital signs monitoring and extraction of physiological signals from environmental noise. One of the biggest challenges of camera-derived monitoring is artefacts from motion, which limits our understanding of what parameters may be derived from skin. In this study, we use phenylephrine and glyceryl trinitrate (GTN) to cause vasoconstriction and vasodilation in stationery healthy volunteers with the aim of describing directional changes in skin perfusion pattern.

Methods and analysis: We aim to recruit 30 healthy volunteers who will undergo protocolised infusions of phenylephrine and GTN, followed by monitored and timed release of a thigh tourniquet. The experimental timeline will be identical for all participants. Measurements of traditionally used haemodynamic markers (heart rate, blood pressure, stroke volume) and camera-derived measurements will be taken concurrently throughout the experimental period. The parameters of interest from the image data are skin colour and pattern, skin surface temperature, pulsatile signal detected at skin surface, and skin perfusion index.

Ethics and dissemination: This study was reviewed and approved by the Oxford University Research and Ethics Committee and Clinical Trials and Research Governance teams (R63796/RE001). The results of this study will be presented at scientific conferences and published in peer-reviewed journals.

Trial registration number: ISCTRN10417167

For peer review only

Strengths and limitations of this study

- Our novel study design within the field of optical monitoring allows description of the chronological order and pattern of leg perfusion at skin surface level.
- Using pharmacological challenges will allow us to answer definitively whether physiological signals of interest are present at skin level without artefacts created by movement.
- There is strong physiological and anatomical reasoning to believe that skin perfusion can be a sensitive marker of cardiovascular collapse compared to other traditionally used haemodynamic markers such as urine output or Glasgow Coma Scale (GCS).
- The main limitation of the study is that it is performed in a healthy cohort of participants, rather than in the population of interest (critically unwell). However, studying what happens in health may allow us to determine the normative patterns in the parameters of interest and find out what is abnormal in critical illness in the future. We will construct mapping of lower limb perfusion in the healthy population as a foundation for further studies in the critically ill population.

For peer review only

Introduction

Clinical examination adds valuable information to the overall haemodynamic assessment of a critically unwell patient [1]. The pattern of distribution of blood to the body can assist assessment of the global circulation [2]. As an organ, the perfusion of skin can indirectly portray the functional status of the cardiovascular system [1,3,4], akin to the use of urine output to demonstrate adequate renal perfusion during haemodynamic assessment. This is true for assessing the response to specific rescue therapies [5,6] as well as during the initial clinical assessment. As a less privileged site, skin offers a unique target for non-invasive monitoring where deterioration may be picked up at an early point to allow intervention. This idea is appealing as we know that by the time many other organs display signs of ischaemia, this transient period of localised hypoxia has implications long after the haemodynamic compromise is resolved (e.g. the long-term effect of acute kidney injury explored by Doyle et al. [7] and the short and long-term consequences of acute liver failure due to ischaemia discussed in Taylor et al. [8]). The ease of access to the skin also provides rationale for exploring its use as a monitoring target.

Recent efforts in camera-derived non-contact monitoring have identified several parameters of interest that can be measured at the skin surface [9]. In the visible spectrum, image photoplethysmography (PPG) allows measurement of beat-to-beat information, although the precise origin of this fluctuation measured at the skin surface is still not clear [10]. Thermal camera measurements in the long wave infrared range traditionally targeted nasal temperature fluctuation to estimate the respiratory rate, but there have also been attempts to use surface temperature gradient to estimate haemodynamic status [11]. Laser speckle contrast imaging (LSCI) is a technique that allows measurement of the flow of blood in the vessels immediately under the skin surface [12], which can be achieved using laser within or outside the visible spectrum. LSCI's current use is mainly in imaging the cerebral blood flow, wound assessment, and intra-operative vessel monitoring. Imaging within these different spectral ranges provide diverse information, as they will penetrate skin surface to a variable degree [13].

To date, efforts made in this field have used the clinically trained eye as the definitive model monitor. However, methods based on the function of our eyes are often limited to the visible wavelength spectrum. As our vision relies on the visible spectrum, this methodology allows minimal depth penetration. Other monitors used frequently in clinical practice use other spectral ranges. For example, oxygen carriage in the blood is measured by comparing absorption of light corresponding to wavelengths of 660nm (red) and 940nm (infrared). Furthermore, the clinician uses multiple parameters to make an overall assessment of the haemodynamic status such as skin temperature, skin colour, peripheral pulses, and clamminess [14]. Rather than relying on the accuracy and reliability of signal measured in one wavelength alone, the use of multiple parameters measured across a larger wavelength spectrum to reach a composite conclusion similar to our clinical practice may provide richer information about the circulatory state.

Movement artefact remain a key challenge in image-derived measurements [9], even when the parameter of interest is technically relatively accessible (e.g. heart rate or respiratory rate). In our previous study, we saw that exercise-induced 'physiological' cardiovascular changes created a significant degree of movement artefact that it was not possible to assess for certain whether skin carries a meaningful signal that could be detected during these periods.

For peer review only

This study aims to describe the dynamic skin response to induced changes in haemodynamics, using a range of non-contact cameras. Following the description of baseline perfusion pattern at the skin surface, we will then create controlled changes by giving escalating infusions of phenylephrine and glyceryl trinitrate (GTN) with the aim of constricting and dilating the surface vessels respectively. Using these controlled pharmacological methods, we aim to induce variations in skin perfusion and assess the pattern change while the amount of blood under the skin changes in opposite directions. All subjects will undergo the same study protocol without randomisation. This protocol follows the guidelines from SPIRIT 2013 Statement [15].

For peer review only

Methods and analysis

We aim to recruit 30 healthy subjects aged between 18 and 65 years. As this is an unexplored field, the effect size is not known and it is not possible to calculate a sample size. Therefore this number is based on a balance of adequate study size to describe inter-subject differences and sufficient numbers of males/females and potential skin types being included, with practical considerations of data size for analysis and running of the study. It is expected that the majority of the healthy volunteers will be approached via word of mouth and posters. Details of study inclusion and exclusion criteria are shown in Table 1. In order to improve the quality of the images taken and reduce the amount of noise in the images, participants will be asked to remove lower limb body hair at least 24 hours prior to the study visit. The study will take place at Cardiovascular Clinical Research Facility within John Radcliffe Hospital, Oxford, UK where clinical devices and full resuscitation equipment are available.

Table 1. Inclusion and exclusion criteria

**Inclusion criteria**	
	Healthy adults aged 18 to 65 years
	Willing and able to given informed consent for participation in the study
	Willing to remove hair on lower limbs by shaving or waxing 24 hours prior to the study visit
**Exclusion criteria**	
	Participants whose anatomy, condition, or other required monitoring precludes the use of the camera equipment or thoracic bioimpedance monitor device. Examples include skin disorders such as eczema, scleroderma, or psoriasis
	Any degree of lower limb amputation
	History of surgical intervention to the thigh/knee/lower leg, except for procedures not expected to permanently change blood flow pattern to the lower limb skin (e.g. knee arthroscopy)
	Allergy to silver chloride ECG sensors
	Hyperthyroidism (intravenous phenylephrine contraindicated)
	Any regular medication except oral contraception
	Pregnant or breastfeeding
	History or current neurological condition affecting peripheral circulation
	History of cardiovascular disease where phenylephrine or glyceryl trinitrate are contraindicated
	History of severe headaches

Experimental timeline

Each study visit will begin with informed consent taken by the lead clinician, a final eligibility check, a cardiovascular examination (to exclude any conditions making the pharmacological challenges unsuitable or unsafe), measurement of height, weight, Fitzpatrick skin scale, and baseline vital signs observations including heart rate, blood pressure, respiratory rate, and oxygen saturation.

For peer review only

Following intravenous cannulation of the right upper limb, participants will be asked to remain supine. All monitoring equipment (including reference monitors and camera equipment) will be attached to the participants and electronically time-synchronised. The cameras will record the skin of right lower limb only. Data from the cameras will be stored in encrypted portable data storage and transferred to secure university server as soon as possible.

The experimental period will begin with a 5-minute rest with no interventions. After 5 minutes, we will administer phenylephrine hydrochloride intravenously over a 15-minute period with increase in dose every minute for the first 10 minutes. The planned dose escalation is described in subsection 'Drug infusion protocol'. Subjects will then be given a rest period of 20 minutes to allow washout of the phenylephrine. Following the washout period, we will administer GTN intravenously over a 15-minute period with increase in dose every minute for the first 10 minutes as per protocol. After another 20-minute washout period, we will study re-filling pattern of lower limb using a thigh tourniquet. We will use a Hemaclear (OHK Medical Devices, Haifa, Israel) to achieve exanguination of the right lower limb and as a right thigh tourniquet. The tourniquet will remain in place for 1 minute before being removed while we monitor the limb using the experimental and reference monitors.

Participants and researchers will not be blinded to treatment being given.

Participation in the study is voluntary and it will be made clear to the participants during the informed consent process that they may withdraw from the study at any time during the study visit without citing a reason. In cases of early cessation of the study, the researchers will discuss with each participant whether they wish for the study recording to be deleted. Side effect experienced during the drug experiment will not automatically lead to the participant being withdrawn. After a recovery period of up to 30 minutes, participants may proceed to any remaining study parts if both the participant and the lead clinician are in agreement.

Reference measurements

- Standard non-invasive blood pressure monitor using an inflating cuff, placed on the right upper limb.
- Thoracic bioimpedance monitor to estimate stroke volume. The ancillary data (electrocardiogram, impedance waveform and derived measures including acceleration index, velocity index, pre-ejection period and conventional pulse transit time) will also be recorded.
- A pulse transit time monitor which integrates R-wave estimate, PPG, and chest wall inductance monitoring.

Camera equipment

- Red-Green-Blue (RGB) camera (Grasshopper3 GS3-U3-51S5C, FLIR Systems Inc, Oregon, USA) – visible spectrum, three channel, 60 frames per second (drug infusion periods) or 140 frames per second (tourniquet release);
- Commercial thermographic camera (A6750sc, FLIR Systems Inc, Oregon, USA) – thermal spectrum, 60 frames per second;

For peer review only

- Commercial LSCI (moorFLPI-2, Moor Instruments, Axminster, UK) with near infrared laser diode 785nm. Camera resolution 580 x 752 pixels, scan area 15cm x 20cm, 5 frames per second (drug infusion periods) or 25 frames per second (tourniquet release).

Data monitoring

A dataset will be defined as 'full' when the following are available for the participant:

- Collection of background characteristics: height, weight, Fitzpatrick skin scale, and baseline vital signs observations including heart rate, blood pressure, respiratory rate, and oxygen saturation
- RGB camera data to include periods of phenylephrine infusion, GTN infusion, and timed tourniquet release
- Commercial thermographic camera data to include periods of phenylephrine infusion, GTN infusion, and timed tourniquet release
- Commercial LSCI data to include periods of phenylephrine infusion, GTN infusion, and timed tourniquet release
- Reference measurements (blood pressure at 1-minute interval, stroke volume, ECG, impedance waveform, acceleration index, velocity index, pre-ejection period and conventional pulse transit time, R-wave estimate, peripheral PPG, and chest wall inductance monitoring) to include periods of phenylephrine infusion, GTN infusion, and timed tourniquet release

Any incomplete dataset will not count towards the target of 30 participants. The recording of the above dataset will be checked by the researchers during the study visit in real time and immediately post study visit prior to transfer to permanent storage.

Completeness of dataset and any unforeseen problems arising from the study will be discussed in weekly study management meetings between MH, CA, JJ, and MV or in study supervision meetings between MH, JDY, LT, and PW.

Drug infusion protocol

Phenylephrine infusion will be prepared by mixing phenylephrine chloride with 0.9% sodium chloride solution to create a concentration of 100mcg/ml. The prepared solution will be drawn up into a 50ml syringe. The infusion rate will be started at 0.2mcg/kg/min and increased by 0.2mcg/kg/min increments every 1 minute. No further increases will be made once a 30% increase in mean arterial pressure is reached. Maximum infusion rate indexed to actual body weight will be set at 2mcg/kg/min and once this rate is reached the infusion and all monitoring will continue for further 5 minutes. The maximum absolute initial infusion rate will be set at 14mcg/min, and the maximum absolute peak infusion rate will be 140mcg/min.

Pre-prepared GTN solution at concentration of 1mg/ml will be used for the study. The solution will be drawn up into a 20ml syringe. The infusion will be started at 0.15mcg/kg/min and increased by 0.15mcg/kg/min every 1 minute. No further increases will be made once a 30% fall in mean arterial pressure is reached. Maximum infusion rate indexed to actual body weight allowed will be set at 1.5mcg/kg/min and once this rate is reached the infusion and all monitoring will continue for further 5 minutes. Both drugs will be delivered using an infusion pump connected to the intravenous

For peer review only

cannula using a two port connector. The maximum absolute initial infusion rate will be set at 10.5mcg/min, and the maximum absolute peak infusion rate will be 105mcg/min.

Any side effects reported by participants will lead to immediate reduction of the drug dose to the immediate preceding infusion rate and documentation of the effect on participant study record.

Data collection and statistical analysis

The haemodynamic variables measured using reference devices will be continuously recorded and analysed offline. The LSCI monitor results will be collected using manufacturer's own software (Windows based control, image processing and analysis) and exported to Matlab for analysis. All images collected via the RGB and thermal cameras will be saved onto portable hard drive using purpose-built software and analysed offline.

The main outcome measures in this study are the data collected using video cameras. Three non-contact parameters will be measured from the three cameras. From the RGB camera, the measurement of interest will be the remote PPG signal across the region of interest (ROI) and the gradient between high and low signal amplitude areas. From the thermal camera, measurements of radiance from ROI will be measured. From the LSCI, three ROI will be selected manually to be centred over the knee, middle of the thigh, and lower leg. For each ROI, average flux (manufacturer estimation of blood velocity) will be measured.

For each parameter taken from the cameras, we will compare the difference between baseline (rest period prior to drug challenge) and the peak effect (final period of the drug challenge). We will use an appropriate statistical test to show whether a significant shift in parameters occur with each drug infusion. All values taken throughout each phase of the experimental period will be plotted for qualitative description of the effect of increasing each drug dose. The plots will be used to show pattern of effect of vasoconstriction and vasodilation on the different parameters.

Other parameters that will be measured from the camera images will include pulse transit estimation from PPG signal between proximal and distal ROI, surface temperature gradient between proximal and distal ROI, proportion of visible skin surface with pulsatile component, and colour changes with increasing phenylephrine and GTN infusion.

The timed tourniquet release will be monitored using the same triple camera set up. The outcome of interest from this phase is the regional pattern in reperfusion, with the particular aim of identifying the chronological order of reperfusion of different skin regions. This will be noted from colour and skin surface temperature changes, remote PPG appearance in segmented ROIs, and flux changes. Any early filling areas identified will be labelled as potential arterial perforator vessel site and overlaid on images from the drug infusion phase of the study.

The data will be presented as mean +/-SD unless otherwise stated. P-values < 0.05 were considered statistically significant.

For peer review only

Ethics and dissemination

This study was reviewed and approved by the Oxford University Research and Ethics Committee and Clinical Trials and Research Governance teams (R63796/RE001). The study is registered with ISRTN registry (ISRCTN10417167). Participants will be sent a detailed participant information sheet and given at least 24 hours for consideration whether to partake in the study. The participant information sheet and the recruitment poster have been reviewed and approved by the ethics committee for complete and fair description of taking part. We will obtain written informed consent from all participants. Any protocol amendments will be submitted to the Oxford University Research and Ethics Committee and Clinical Trials and Research Governance teams for approval before being incorporated.

The findings from the study may be disseminated using academic media including peer-reviewed journal articles, national and international conference presentations, social media (including Twitter), electronic mail within the University of Oxford, and the internet including the departmental websites. The findings from the study will form part of a doctoral dissertation for MH. Authorship of journal articles related to this study will follow the ICMJE guidelines [16]. Due to the nature of the data being collected (video/image-based), it is impossible to completely anonymise the data source. This will be explicitly discussed during the informed consent. Participants will be identified only by a participant identification number on study documents and any electronic database. Documents that contain identifying data and/or information allowing linkage between participant identification number and personal data will be stored separately under strict access controls. The participants will retain the right to have their images deleted from storage at any time upon request.

Access to data

The data, stored in a proprietary format that is only readable by the review software, will be recorded onto an encrypted portable storage device that will be transferred by the researchers in secured storage to the University server. Except during transport, access to the data will be restricted to academic areas behind two limited access doors. All study documents will be stored securely and only accessible by study staff (Kadoorie Centre for Critical Care Research and Education, and the Institute of Biomedical Engineering/Oxford University Centre of Excellence for Medical Engineering) and authorised personnel from the University of Oxford for monitoring and/or audit of the study to ensure compliance with regulations. All video data will be stored securely using industry-standard encryption methods. The video data forms part of the research data and will be stored securely for 5 years after the release date of the last publication arising from the study.

Ancillary and post-trial care

If a participant in this study is ever considered to have suffered harm through their participation in the study, the University of Oxford as the sponsor has arrangements to provide compensation. Participants will be informed of potential route for escalation of any issues that arise from the study via the study team or via the Chair of the Medical Sciences Interdivisional Research Ethics Committee (MS IDREC) at the University of Oxford as appropriate.

For peer review only

Discussion

To the best of our knowledge, this is the first study aiming to create a skin surface perfusion map of the lower limbs and study changes to the map with increase and decrease in peripheral skin blood flow. This is one of a series of planned studies designed to answer how much information can be measured at accessible skin surface level using non-contact tools.

A limitation of this proposed study is that it is only including healthy volunteers in artificial controlled conditions with restricted movement. However, we feel that this is an important initial step as if no useful signal can be derived from the best possible environment with no movement artefacts, further efforts at looking for this absent signal in more realistic situations may not be fruitful. Creating optimum conditions by limiting movement will allow us to isolate signals that are present and potentially allow a better understanding of these signals of interest so that they can be isolated more effectively within noisy environment. Some concerns may be raised by the use of healthy population. The haemodynamic control requires a fine balance of our sympathetic and parasympathetic systems, and we know that this balance is not at baseline in the critically ill. However, the understanding of how the differing physiology in healthy and non-healthy populations translate to changes measured at skin surface will expand our understanding of critical illness and haemodynamic collapse.

Another key challenge is that by using specific receptor agonists, we bypass the physiological pathway by which the skin blood flow is reduced or increased. In healthy population used in the proposed study, we expect the vessel changes to cause subsequent changes in central cardiovascular balance. In critical illness, haemodynamic collapse and low blood pressure leads to peripheral vasoconstriction and reduced peripheral circulation. Creating the same peripheral conditions using phenylephrine infusion will lead to a rise in blood pressure. However, our methodology will allow us to see the pattern of reduction and increase in skin perfusion. This will provide invaluable information when similar changes are tracked on skin surface in patient populations.

For peer review only

References

1. van Genderen ME, Paauwe J, de Jonge J, *et al*. Clinical assessment of peripheral perfusion to predict postoperative complications after major abdominal surgery early: a prospective observational study in adults. *Crit Care* 2014;18(3):R114–R114.
2. Calzia E, Iványi Z, Radermacher P. Determinants of blood flow and organ perfusion. In: Functional hemodynamic monitoring. Springer 2005:19–32.
3. Van Genderen ME, Bartels SA, Lima A, *et al*. Peripheral perfusion index as an early predictor for central hypovolemia in awake healthy volunteers. *Anesth Analg* 2013;116(2):351–356.
4. Vazquez R, Vazquez Guillamet C, Adeel Rishi M, *et al*. Physical examination in the intensive care unit: opinions of physicians at three teaching hospitals. *Southwest J Pulm Crit Care* 2015;10(1):34–43.
5. Lima A, Jansen TC, van Bommel J, *et al*. The prognostic value of the subjective assessment of peripheral perfusion in critically ill patients. *Crit Care Med* 2009;37(3):934–938.
6. Lima A, Bakker J. Clinical monitoring of peripheral perfusion: there is more to learn. *Crit Care* 2014;18(1):113.
7. Doyle JF, Forni LG. Acute kidney injury: short-term and long-term effects. *Crit Care* 2016;20(1):188.
8. Taylor RM, Tujios S, Jinjuvadia K, *et al*. Short and long-term outcomes in patients with acute liver failure due to ischemic hepatitis. *Dig Dis Sci* 2012;57(3):777–785.
9. Harford M, Catherall J, Gerry S, *et al*. Availability and performance of image-based, non-contact methods of monitoring heart rate, blood pressure, respiratory rate, and oxygen saturation: A systematic review. *Physiol Meas* 2019;40(6):06TR01
10. Sun Y, Thakor N. Photoplethysmography Revisited: From Contact to Noncontact, From Point to Imaging. *IEEE Trans Biomed Eng* 2016;63(3):463–477.
11. Cooke WH, Moralez G, Barrera CR, *et al*. Digital infrared thermographic imaging for remote assessment of traumatic injury. *J Appl Physiol (1985)* 2011;111(6):1813–1818.
12. Richards LM, Kazmi SM, Davis JL, *et al*. Low-cost laser speckle contrast imaging of blood flow using a webcam. *Biomed Opt Express* 2013;4(10):2269–83.
13. Ash C, Dubec M, Donne K, *et al*. Effect of wavelength and beam width on penetration in light-tissue interaction using computational methods. *Lasers Med Sci* 2017;32(8):1909–1918.
14. Dünser MW, Dankl D, Petros S, *et al*. Clinical Examination Skills in the Adult Critically Ill Patient. Springer; 2018.
15. Chan A-W, Tetzlaff JM, Altman DG, *et al*. SPIRIT 2013 Statement: Defining standard protocol items for clinical trials. *Ann Intern Med* 2013;158:200-207.
16. Recommendations for the Conduct, Reporting, Editing, and Publication of Scholarly works in Journals. International Committee of Medical journal Editors. Available: http://icmje.org/recommendations [Accessed on 23 July 2019].

For peer review only

Authors' contributions

MH conceived the study with guidance from PW, DY, LT, MV and JJ. MH, CA, MV, JJ, EF, and SD will conduct screening and data collection. MH and AM planned the statistical analysis. Analysis will be performed by MH, MV, JJ, EF and SD. MH prepared the first draft of this manuscript. All authors critiqued and edited the manuscript for intellectual content.

Trial sponsor

University of Oxford, Oxford, United Kingdom

Funding statement

This work was supported by NIHR Oxford Biomedical Research Centre (BRC) under Technology & Digital Health theme. NIHR Oxford BRC advocates open access. MH, CA and PW are funded by NIHR Oxford BRC. The work of JJ was supported by the RCUK Digital Economy Programme, grant number EP/G036861/1 (Oxford Centre for Doctoral Training in Healthcare Innovation). JJ also acknowledges Fundacao para a Ciencia e Tecnologia, Portugal, doctoral grant SFRH/BD/85158/2012. MV was supported by the Oxford Centre of Excellence in Medical Engineering funded by the Wellcome Trust and EPSRC under grant number WT88877/Z/09/Z.

The funders have had no role in the study protocol design or the preparation of this manuscript, and will have no role in the collection, management, analysis and interpretation of the data, or the writing of the final report.

Competing interests statement

PW is Chief Medical Officer for Sensyne Health and holds share options in the company. His organisation has received research funding from Sensyne Health not associated with this work. LT is a non-executive Director of Sensyne Health and holds share options in the company. LT is a non-executive Director of Oxehealth and holds shares in the company.

Patient and Public Involvement

The aims of the study and its potential role in the design of future studies into haemodynamic monitoring within critical care was discussed with a critical care patient group based in Oxford University Hospitals NHS Foundation Trust.

Word count

3456

For peer review only

STANDARD PROTOCOL ITEMS: RECOMMENDATIONS FOR INTERVENTIONAL TRIALS

SPIRIT 2013 Checklist for Mapping Of Lower Limb skin pErfusion (MOLLIE): Recommended items to address in a clinical trial protocol and related documents*

Section/item	Item No	Description	Addressed on page number
**Administrative information**			
Title	1	Descriptive title identifying the study design, population, interventions, and, if applicable, trial acronym	1
Trial registration	2a	Trial identifier and registry name. If not yet registered, name of intended registry	2, 10
	2b	All items from the World Health Organization Trial Registration Data Set	ISRCTN.com
Protocol version	3	Date and version identifier	1
Funding	4	Sources and types of financial, material, and other support	13
Roles and responsibilities	5a	Names, affiliations, and roles of protocol contributors	1, 13
	5b	Name and contact information for the trial sponsor	13
	5c	Role of study sponsor and funders, if any, in study design; collection, management, analysis, and interpretation of data; writing of the report; and the decision to submit the report for publication, including whether they will have ultimate authority over any of these activities	13
	5d	Composition, roles, and responsibilities of the coordinating centre, steering committee, endpoint adjudication committee, data management team, and other individuals or groups overseeing the trial, if applicable (see Item 21a for data monitoring committee)	N/A

For peer review only

Introduction

Background and rationale	6a	Description of research question and justification for undertaking the trial, including summary of relevant studies (published and unpublished) examining benefits and harms for each intervention	4 – 5
	6b	Explanation for choice of comparators	4 – 5
Objectives	7	Specific objectives or hypotheses	5
Trial design	8	Description of trial design including type of trial (eg, parallel group, crossover, factorial, single group), allocation ratio, and framework (eg, superiority, equivalence, noninferiority, exploratory)	5

Methods: Participants, interventions, and outcomes

Study setting	9	Description of study settings (eg, community clinic, academic hospital) and list of countries where data will be collected. Reference to where list of study sites can be obtained	6
Eligibility criteria	10	Inclusion and exclusion criteria for participants. If applicable, eligibility criteria for study centres and individuals who will perform the interventions (eg, surgeons, psychotherapists)	6
Interventions	11a	Interventions for each group with sufficient detail to allow replication, including how and when they will be administered	6 – 7
	11b	Criteria for discontinuing or modifying allocated interventions for a given trial participant (eg, drug dose change in response to harms, participant request, or improving/worsening disease)	7
	11c	Strategies to improve adherence to intervention protocols, and any procedures for monitoring adherence (eg, drug tablet return, laboratory tests)	N/A
	11d	Relevant concomitant care and interventions that are permitted or prohibited during the trial	N/A
Outcomes	12	Primary, secondary, and other outcomes, including the specific measurement variable (eg, systolic blood pressure), analysis metric (eg, change from baseline, final value, time to event), method of aggregation (eg, median, proportion), and time point for each outcome. Explanation of the clinical relevance of chosen efficacy and harm outcomes is strongly recommended	8
Participant timeline	13	Time schedule of enrolment, interventions (including any run-ins and washouts), assessments, and visits for participants. A schematic diagram is highly recommended (see Figure)	5 – 7

For peer review only - http://bmjopen.bmj.com/site/about/guidelines.xhtml

Sample size	14	Estimated number of participants needed to achieve study objectives and how it was determined, including clinical and statistical assumptions supporting any sample size calculations	6
Recruitment	15	Strategies for achieving adequate participant enrolment to reach target sample size	6

Methods: Assignment of interventions (for controlled trials)

Allocation:

Sequence generation	16a	Method of generating the allocation sequence (eg, computer-generated random numbers), and list of any factors for stratification. To reduce predictability of a random sequence, details of any planned restriction (eg, blocking) should be provided in a separate document that is unavailable to those who enrol participants or assign interventions	N/A
Allocation concealment mechanism	16b	Mechanism of implementing the allocation sequence (eg, central telephone; sequentially numbered, opaque, sealed envelopes), describing any steps to conceal the sequence until interventions are assigned	N/A
Implementation	16c	Who will generate the allocation sequence, who will enrol participants, and who will assign participants to interventions	N/A
Blinding (masking)	17a	Who will be blinded after assignment to interventions (eg, trial participants, care providers, outcome assessors, data analysts), and how	N/A
	17b	If blinded, circumstances under which unblinding is permissible, and procedure for revealing a participant's allocated intervention during the trial	N/A

Methods: Data collection, management, and analysis

Data collection methods	18a	Plans for assessment and collection of outcome, baseline, and other trial data, including any related processes to promote data quality (eg, duplicate measurements, training of assessors) and a description of study instruments (eg, questionnaires, laboratory tests) along with their reliability and validity, if known. Reference to where data collection forms can be found, if not in the protocol	7 – 8
	18b	Plans to promote participant retention and complete follow-up, including list of any outcome data to be collected for participants who discontinue or deviate from intervention protocols	7 – 8

Data management	19	Plans for data entry, coding, security, and storage, including any related processes to promote data quality (eg, double data entry; range checks for data values). Reference to where details of data management procedures can be found, if not in the protocol	7 – 9
Statistical methods	20a	Statistical methods for analysing primary and secondary outcomes. Reference to where other details of the statistical analysis plan can be found, if not in the protocol	8 – 9
	20b	Methods for any additional analyses (eg, subgroup and adjusted analyses)	8 – 9
	20c	Definition of analysis population relating to protocol non-adherence (eg, as randomised analysis), and any statistical methods to handle missing data (eg, multiple imputation)	7 – 8
**Methods: Monitoring**			
Data monitoring	21a	Composition of data monitoring committee (DMC); summary of its role and reporting structure; statement of whether it is independent from the sponsor and competing interests; and reference to where further details about its charter can be found, if not in the protocol. Alternatively, an explanation of why a DMC is not needed	10
	21b	Description of any interim analyses and stopping guidelines, including who will have access to these interim results and make the final decision to terminate the trial	N/A
Harms	22	Plans for collecting, assessing, reporting, and managing solicited and spontaneously reported adverse events and other unintended effects of trial interventions or trial conduct	8
Auditing	23	Frequency and procedures for auditing trial conduct, if any, and whether the process will be independent from investigators and the sponsor	10
**Ethics and dissemination**			
Research ethics approval	24	Plans for seeking research ethics committee/institutional review board (REC/IRB) approval	10
Protocol amendments	25	Plans for communicating important protocol modifications (eg, changes to eligibility criteria, outcomes, analyses) to relevant parties (eg, investigators, REC/IRBs, trial participants, trial registries, journals, regulators)	10

For peer review only - http://bmjopen.bmj.com/site/about/guidelines.xhtml

Consent or assent	26a	Who will obtain informed consent or assent from potential trial participants or authorised surrogates, and how (see Item 32)	10
	26b	Additional consent provisions for collection and use of participant data and biological specimens in ancillary studies, if applicable	N/A
Confidentiality	27	How personal information about potential and enrolled participants will be collected, shared, and maintained in order to protect confidentiality before, during, and after the trial	10
Declaration of interests	28	Financial and other competing interests for principal investigators for the overall trial and each study site	13
Access to data	29	Statement of who will have access to the final trial dataset, and disclosure of contractual agreements that limit such access for investigators	10
Ancillary and post-trial care	30	Provisions, if any, for ancillary and post-trial care, and for compensation to those who suffer harm from trial participation	10
Dissemination policy	31a	Plans for investigators and sponsor to communicate trial results to participants, healthcare professionals, the public, and other relevant groups (eg, via publication, reporting in results databases, or other data sharing arrangements), including any publication restrictions	10
	31b	Authorship eligibility guidelines and any intended use of professional writers	10 (ICMJE)
	31c	Plans, if any, for granting public access to the full protocol, participant-level dataset, and statistical code	N/A
**Appendices**			
Informed consent materials	32	Model consent form and other related documentation given to participants and authorised surrogates	Appendix
Biological specimens	33	Plans for collection, laboratory evaluation, and storage of biological specimens for genetic or molecular analysis in the current trial and for future use in ancillary studies, if applicable	N/A

*It is strongly recommended that this checklist be read in conjunction with the SPIRIT 2013 Explanation & Elaboration for important clarification on the items. Amendments to the protocol should be tracked and dated. The SPIRIT checklist is copyrighted by the SPIRIT Group under the Creative Commons "Attribution-NonCommercial-NoDerivs 3.0 Unported" license.

BMJ Open

A study protocol for an exploratory interventional study investigating the feasibility of video-based non-contact physiological monitoring in healthy volunteers by Mapping Of Lower Limb skIn pErfusion (MOLLIE)

Journal:	*BMJ Open*
Manuscript ID	bmjopen-2019-036235.R1
Article Type:	Protocol
Date Submitted by the Author:	17-Mar-2020
Complete List of Authors:	Harford, Mirae; University of Oxford, Nuffield Department of Clinical Neurosciences; University of Oxford, Institute of Biomedical Engineering, Department of Engineering Science<br>Areia, Carlos; University of Oxford, Nuffield Department of Clinical Neurosciences<br>Villarroel, Mauricio; University of Oxford, Institute of Biomedical Engineering, Department of Engineering Science<br>Jorge, Joao; University of Oxford, Institute of Biomedical Engineering, Department of Engineering Science<br>Finnegan, Eoin; University of Oxford, Institute of Biomedical Engineering, Department of Engineering Science<br>Davidson, Shaun; University of Oxford, Institute of Biomedical Engineering, Department of Engineering Science<br>Mahdi, Adam; University of Oxford, Institute of Biomedical Engineering, Department of Engineering Science<br>Young, Duncan; Oxford University, Nuffield Department of Clinical Neurosciences<br>Tarassenko, Lionel; University of Oxford, Institute of Biomedical Engineering, Department of Engineering Science<br>Watkinson, Peter; Oxford University, Nuffield Department of Clinical Neurosciences
**Primary Subject Heading**:	Radiology and imaging
Secondary Subject Heading:	Anaesthesia, Dermatology, Research methods, Cardiovascular medicine, Intensive care
Keywords:	Anatomy < BASIC SCIENCES, CLINICAL PHYSIOLOGY, CLINICAL PHARMACOLOGY

SCHOLARONE™
Manuscripts

I, the Submitting Author has the right to grant and does grant on behalf of all authors of the Work (as defined in the below author licence), an exclusive licence and/or a non-exclusive licence for contributions from authors who are: i) UK Crown employees; ii) where BMJ has agreed a CC-BY licence shall apply, and/or iii) in accordance with the terms applicable for US Federal Government officers or employees acting as part of their official duties; on a worldwide, perpetual, irrevocable, royalty-free basis to BMJ Publishing Group Ltd ("BMJ") its licensees and where the relevant Journal is co-owned by BMJ to the co-owners of the Journal, to publish the Work in this journal and any other BMJ products and to exploit all rights, as set out in our licence.

The Submitting Author accepts and understands that any supply made under these terms is made by BMJ to the Submitting Author unless you are acting as an employee on behalf of your employer or a postgraduate student of an affiliated institution which is paying any applicable article publishing charge ("APC") for Open Access articles. Where the Submitting Author wishes to make the Work available on an Open Access basis (and intends to pay the relevant APC), the terms of reuse of such Open Access shall be governed by a Creative Commons licence – details of these licences and which Creative Commons licence will apply to this Work are set out in our licence referred to above.

Other than as permitted in any relevant BMJ Author's Self Archiving Policies, I confirm this Work has not been accepted for publication elsewhere, is not being considered for publication elsewhere and does not duplicate material already published. I confirm all authors consent to publication of this Work and authorise the granting of this licence.

For peer review only

A study protocol for an exploratory interventional study investigating the feasibility of video-based non-contact physiological monitoring in healthy volunteers by Mapping Of Lower Limb skIn pErfusion (MOLLIE)

Mirae Harford[1,2]

Carlos Areia[1]

Mauricio Villarroel[2]

Joao Jorge[2]

Eoin Finnegan[2]

Shaun Davidson[2]

Adam Mahdi[1,2]

John Duncan Young[1]

Lionel Tarassenko[2]

Peter Watkinson[1]

1. Critical Care Research Group, Kadoorie Centre for Critical Care Research and Education, Nuffield Department of Clinical Neurosciences, University of Oxford, Oxford, United Kingdom
2. Institute of Biomedical Engineering, Department of Engineering Science, University of Oxford, Oxford, United Kingdom

Corresponding author: Mirae Harford (mirae.harford@ndcn.ox.ac.uk), 01865 332101

Word count: 3540

For peer review only

Abstract

Introduction: Skin perfusion varies in response to changes in the circulatory status. Blood flow to skin is reduced during haemodynamic collapse secondary to peripheral vasoconstriction, while increased skin perfusion is frequently observed when haemodynamics improve with resuscitation. These changes in perfusion may be monitored using non-contact image-based methods. Previous camera-derived physiological measurements have focused on accurate vital signs monitoring and extraction of physiological signals from environmental noise. One of the biggest challenges of camera-derived monitoring is artefacts from motion, which limits our understanding of what parameters may be derived from skin. In this study, we use phenylephrine and glyceryl trinitrate (GTN) to cause vasoconstriction and vasodilation in stationery healthy volunteers with the aim of describing directional changes in skin perfusion pattern.

Methods and analysis: We aim to recruit 30 healthy volunteers who will undergo protocolised infusions of phenylephrine and GTN, followed by monitored and timed release of a thigh tourniquet. The experimental timeline will be identical for all participants. Measurements of traditionally used haemodynamic markers (heart rate, blood pressure, stroke volume) and camera-derived measurements will be taken concurrently throughout the experimental period. The parameters of interest from the image data are skin colour and pattern, skin surface temperature, pulsatile signal detected at skin surface, and skin perfusion index.

Ethics and dissemination: This study was reviewed and approved by the Oxford University Research and Ethics Committee and Clinical Trials and Research Governance teams (R63796/RE001). The results of this study will be presented at scientific conferences and published in peer-reviewed journals.

Trial registration number: ISCTRN10417167

For peer review only

Strengths and limitations of this study

- Our novel study design within the field of optical monitoring allows description of the chronological order and pattern of leg perfusion at skin surface level.
- Using pharmacological challenges will allow us to answer definitively whether physiological signals of interest are present at skin level without artefacts created by movement.
- There is strong physiological and anatomical reasoning to believe that skin perfusion can be a sensitive marker of cardiovascular collapse compared to other traditionally used haemodynamic markers such as urine output or Glasgow Coma Scale (GCS).
- This study is limited by the use of healthy subjects to determine the normative patterns in the parameters of interest, rather than monitoring the ultimate population of interest (critically unwell).

For peer review only

Introduction

Clinical examination adds valuable information to the overall haemodynamic assessment of a critically unwell patient [1]. The pattern of distribution of blood to the body can assist assessment of the global circulation [2]. As an organ, the perfusion of skin can indirectly portray the functional status of the cardiovascular system [1,3,4], akin to the use of urine output to demonstrate adequate renal perfusion during haemodynamic assessment. This is true for assessing the response to specific rescue therapies [5,6] as well as during the initial clinical assessment. As a less privileged site, skin offers a unique target for non-invasive monitoring where deterioration may be picked up at an early point to allow intervention. The ease of access to the skin also provides rationale for exploring its use as a monitoring target.

Recent efforts in camera-derived non-contact monitoring have identified several parameters of interest that can be measured at the skin surface [7]. In the visible spectrum, image photoplethysmography (PPG) allows measurement of beat-to-beat information, although the precise origin of this fluctuation measured at the skin surface is still not clear [8]. Thermal camera measurements in the long wave infrared range traditionally targeted nasal temperature fluctuation to estimate the respiratory rate, but there have also been attempts to use surface temperature gradient to estimate haemodynamic status [9]. Laser speckle contrast imaging (LSCI) is a technique that allows measurement of the flow of blood in the vessels immediately under the skin surface [10], which can be achieved using laser within or outside the visible spectrum. LSCI's current use is mainly in imaging the cerebral blood flow, wound assessment, and intra-operative vessel monitoring. Imaging within these different spectral ranges provide diverse information, as they will penetrate skin surface to a variable degree [11].

To date, efforts made in this field have used the clinically trained eye as the definitive model monitor. However, methods based on the function of our eyes are often limited to the visible wavelength spectrum. As our vision relies on the visible spectrum, this methodology allows minimal depth penetration. Other monitors used frequently in clinical practice use other spectral ranges. For example, oxygen carriage in the blood is measured by comparing absorption of light corresponding to wavelengths of 660nm (red) and 940nm (infrared). Furthermore, the clinician uses multiple parameters to make an overall assessment of the haemodynamic status such as skin temperature, skin colour, peripheral pulses, and clamminess [12]. Rather than relying on the accuracy and reliability of signal measured in one wavelength alone, the use of multiple parameters measured across a larger wavelength spectrum to reach a composite conclusion similar to our clinical practice may provide richer information about the circulatory state.

Movement artefact remain a key challenge in image-derived measurements [9], even when the parameter of interest is technically relatively accessible (e.g. heart rate or respiratory rate). In our previous study, we saw that exercise-induced 'physiological' cardiovascular changes created a significant degree of movement artefact that it was not possible to assess for certain whether skin carries a meaningful signal that could be detected during these periods.

This study aims to describe the dynamic skin response to induced changes in haemodynamics, using a range of non-contact cameras. Following the description of baseline perfusion pattern at the skin surface, we will then create controlled changes by giving escalating infusions of phenylephrine and glyceryl trinitrate (GTN) with the aim of constricting and dilating the surface vessels respectively. Using these controlled pharmacological methods, we aim to induce variations in skin perfusion and

For peer review only

assess the pattern change while the amount of blood under the skin changes in opposite directions. We hypothesise that the induced increase and decrease in skin perfusion (and the resultant changes in skin colour, surface temperature, blood flow velocity) may be detected non-invasively using cameras detecting signals within an outside the visible spectrum. The expected changes and the cameras being used to detect the changes are shown in Table 1. In order to describe these changes with detailed knowledge of the lower limb vascular anatomy, we plan to use an arterial tourniquet and timed release to identify the arterial perforator vessel locations [13] that represent the dominant branch supplying each region of skin. All subjects will undergo the same study protocol without randomisation. This protocol follows the guidelines from SPIRIT 2013 Statement [14].

Table 1. Expected changes with planned infusions

	**Camera detecting changes**	**Expected changes with infusions**	
		**Phenylephrine**	**Glyceryl trinitrate**
**Skin colour**	Red/Green/Blue visible spectrum camera	Pale skin, reduced red tone with vessel constriction	Increase in red tone with vessel dilation
**Skin temperature**	Thermographic camera	Reduced temperature and thermal signal	Increased temperature and thermal signal
**Skin perfusion**	Laser speckle contrast imager	Reduced perfusion/flux	Increased perfusion/flux
**Pulsatility**	Red/Green/Blue visible spectrum camera	Reduced pulsatility in image plethysmography	Increased pulsatility in image plethysmography

For peer review only

Methods and analysis

We aim to recruit 30 healthy subjects aged between 18 and 65 years, aiming for a balanced ratio of male to female participants. As this is an unexplored field, the effect size is not known and it is not possible to calculate a sample size. Therefore this number is based on a balance of adequate study size to describe inter-subject differences and sufficient numbers of males/females and potential skin types being included, with practical considerations of data size for analysis and running of the study. It is expected that the majority of the healthy volunteers will be approached via word of mouth and posters. Details of study inclusion and exclusion criteria are shown in Table 2. In order to improve the quality of the images taken and reduce the amount of noise in the images, participants will be asked to remove lower limb body hair at least 24 hours prior to the study visit. The study will take place at Cardiovascular Clinical Research Facility within John Radcliffe Hospital, Oxford, UK where clinical devices and full resuscitation equipment are available.

Table 2. Inclusion and exclusion criteria

**Inclusion criteria**	
	Healthy adults aged 18 to 65 years
	Willing and able to given informed consent for participation in the study
	Willing to remove hair on lower limbs by shaving or waxing 24 hours prior to the study visit
**Exclusion criteria**	
	Participants whose anatomy, condition, or other required monitoring precludes the use of the camera equipment or thoracic bioimpedance monitor device. Examples include skin disorders such as eczema, scleroderma, or psoriasis
	Any degree of lower limb amputation
	History of surgical intervention to the thigh/knee/lower leg, except for procedures not expected to permanently change blood flow pattern to the lower limb skin (e.g. knee arthroscopy)
	Allergy to silver chloride ECG sensors
	Hyperthyroidism (intravenous phenylephrine contraindicated)
	Any regular medication except oral contraception
	Pregnant or breastfeeding
	History or current neurological condition affecting peripheral circulation
	History of cardiovascular disease where phenylephrine or glyceryl trinitrate are contraindicated
	History of severe headaches

Experimental timeline

Each study visit will begin with informed consent taken by the lead clinician and a final eligibility check. A cardiovascular examination will be performed including an examination of the peripheries for signs of cardiovascular disease, palpation of central pulse, and auscultation of heart sounds (to exclude any conditions making the pharmacological challenges unsuitable or unsafe). We will also measure participants' height, weight, Fitzpatrick skin scale, and baseline vital signs observations including heart rate, blood pressure, respiratory rate, and oxygen saturation. Participants with

For peer review only

baseline systolic blood pressure above 140 mmHg or baseline diastolic blood pressure above 90 mmHg on repeat measurements will not be included in the study.

Following intravenous cannulation of the right upper limb, participants will be asked to remain supine without movement. Movement of the lower limbs will be limited by a foot rest limiting involuntary external rotation of the leg. All monitoring equipment (including reference monitors and camera equipment) will be attached to the participants and electronically time-synchronised. The cameras will record the skin of right lower limb only. Data from the cameras will be stored in encrypted portable data storage and transferred to secure university server as soon as possible.

The experimental period will begin with a 5-minute rest with no interventions. After 5 minutes, we will administer phenylephrine hydrochloride intravenously over a 15-minute period with increase in dose every minute for the first 10 minutes. The planned dose escalation is described in subsection 'Drug infusion protocol'. Subjects will then be given a rest period of 20 minutes to allow washout of the phenylephrine. Following the washout period, we will administer GTN intravenously over a 15-minute period with increase in dose every minute for the first 10 minutes as per protocol. After another 20-minute washout period, we will study re-filling pattern of lower limb using a thigh tourniquet. We will use a Hemaclear (OHK Medical Devices, Haifa, Israel) to achieve exanguination of the right lower limb and as a right thigh tourniquet. The tourniquet will remain in place for 1 minute before being removed while we monitor the limb using the experimental and reference monitors.

Participants and researchers will not be blinded to treatment being given.

Participation in the study is voluntary and it will be made clear to the participants during the informed consent process that they may withdraw from the study at any time during the study visit without citing a reason. In cases of early cessation of the study, the researchers will discuss with each participant whether they wish for the study recording to be deleted. Side effect experienced during the drug experiment will not automatically lead to the participant being withdrawn. After a recovery period of up to 30 minutes, participants may proceed to any remaining study parts if both the participant and the lead clinician are in agreement.

Reference measurements

- Standard non-invasive blood pressure monitor using an inflating cuff, placed on the left upper limb.
- Thoracic bioimpedance monitor to estimate stroke volume. The ancillary data (electrocardiogram, impedance waveform and derived measures including acceleration index, velocity index, pre-ejection period and conventional pulse transit time) will also be recorded.
- A pulse transit time monitor which integrates R-wave estimate, PPG, and chest wall inductance monitoring.

Camera equipment

- Red-Green-Blue (RGB) camera (Grasshopper3 GS3-U3-51S5C, FLIR Systems Inc, Oregon, USA) – visible spectrum, three channel, 60 frames per second (drug infusion periods) or 140 frames per second (tourniquet release);

For peer review only

- Commercial thermographic camera (A6750sc, FLIR Systems Inc, Oregon, USA) – thermal spectrum, 60 frames per second;
- Commercial LSCI (moorFLPI-2, Moor Instruments, Axminster, UK) with near infrared laser diode 785nm. Camera resolution 580 x 752 pixels, scan area 15cm x 20cm, 5 frames per second (drug infusion periods) or 25 frames per second (tourniquet release).

Data monitoring

A dataset will be defined as 'full' when the following are available for the participant:

- Collection of background characteristics: height, weight, Fitzpatrick skin scale, and baseline vital signs observations including heart rate, blood pressure, respiratory rate, and oxygen saturation
- RGB camera data to include periods of phenylephrine infusion, GTN infusion, and timed tourniquet release
- Commercial thermographic camera data to include periods of phenylephrine infusion, GTN infusion, and timed tourniquet release
- Commercial LSCI data to include periods of phenylephrine infusion, GTN infusion, and timed tourniquet release
- Reference measurements (blood pressure at 1-minute interval, stroke volume, ECG, impedance waveform, acceleration index, velocity index, pre-ejection period and conventional pulse transit time, R-wave estimate, peripheral PPG, and chest wall inductance monitoring) to include periods of phenylephrine infusion, GTN infusion, and timed tourniquet release

Any incomplete dataset will not count towards the target of 30 participants. The recording of the above dataset will be checked by the researchers during the study visit in real time and immediately post study visit prior to transfer to permanent storage.

Completeness of dataset and any unforeseen problems arising from the study will be discussed in weekly study management meetings between MH, CA, JJ, and MV or in study supervision meetings between MH, JDY, LT, and PW.

Drug infusion protocol

Phenylephrine infusion will be prepared by mixing phenylephrine chloride with 0.9% sodium chloride solution to create a concentration of 100mcg/ml. The prepared solution will be drawn up into a 50ml syringe. The infusion rate will be started at 0.2mcg/kg/min and increased by 0.2mcg/kg/min increments every 1 minute. No further increases will be made once a 30% increase in mean arterial pressure is reached. Maximum infusion rate indexed to actual body weight will be set at 2mcg/kg/min and once this rate is reached the infusion and all monitoring will continue for further 5 minutes. The maximum absolute initial infusion rate will be set at 14mcg/min, and the maximum absolute peak infusion rate will be 140mcg/min.

Pre-prepared GTN solution at concentration of 1mg/ml will be used for the study. The solution will be drawn up into a 20ml syringe. The infusion will be started at 0.15mcg/kg/min and increased by 0.15mcg/kg/min every 1 minute. No further increases will be made once a 30% fall in mean arterial pressure is reached. Maximum infusion rate indexed to actual body weight allowed will be set at

For peer review only

1.5mcg/kg/min and once this rate is reached the infusion and all monitoring will continue for further 5 minutes. Both drugs will be delivered using an infusion pump connected to the intravenous cannula using a two port connector. The maximum absolute initial infusion rate will be set at 10.5mcg/min, and the maximum absolute peak infusion rate will be 105mcg/min.

Any side effects reported by participants will lead to immediate reduction of the drug dose to the immediate preceding infusion rate and documentation of the effect on participant study record.

Data collection and statistical analysis

The haemodynamic variables measured using reference devices will be continuously recorded and analysed offline. The LSCI monitor results will be collected using manufacturer's own software (Windows based control, image processing and analysis) and exported to Matlab for analysis. All images collected via the RGB and thermal cameras will be saved onto portable hard drive using purpose-built software and analysed offline.

The main outcome measures in this study are the data collected using video cameras. Three non-contact parameters will be measured from the three cameras and the skin perfusion pattern will be described using composite measures from three different cameras. From the RGB camera, the measurement of interest will be the remote PPG signal across the region of interest (ROI) and the gradient between high and low signal amplitude areas. From the thermal camera, measurements of radiance from ROI will be measured. From the LSCI, three ROI will be selected manually to be centred over the knee, middle of the thigh, and lower leg. For each ROI, average flux (manufacturer estimation of blood velocity) will be measured.

For each parameter taken from the cameras, we will compare the difference between baseline (rest period prior to drug challenge) and the peak effect (final period of the drug challenge). All values taken throughout each phase of the experimental period will be plotted for qualitative description of the effect of increasing each drug dose. The plots will be used to show pattern of effect of vasoconstriction and vasodilation on the different parameters. We will use a paired t-test to test, at the significance level of 0.05, the null hypothesis that there is no difference between the means of baseline and peak effect of the drug challenge. If the assumption of the test are not met by the data, we will use a non-parametric Wilcoxon-Mann-Whitney test instead.

Other parameters that will be measured from the camera images will include pulse transit estimation from PPG signal between proximal and distal ROI, surface temperature gradient between proximal and distal ROI, proportion of visible skin surface with pulsatile component, and colour changes with increasing phenylephrine and GTN infusion.

The timed tourniquet release will be monitored using the same triple camera set up. The outcome of interest from this phase is the regional pattern in reperfusion, with the particular aim of identifying the chronological order of reperfusion of different skin regions. The reperfusion pattern will be described from colour and skin surface temperature changes, remote PPG appearance in segmented ROIs, and flux changes. Any early filling areas identified will be labelled as potential arterial perforator vessel site and overlaid on images from the drug infusion phase of the study.

The data will be presented as mean +/-SD unless otherwise stated. P-values < 0.05 were considered statistically significant.

For peer review only

Ethics and dissemination

This study was reviewed and approved by the Oxford University Research and Ethics Committee and Clinical Trials and Research Governance teams (R63796/RE001). The study is registered with ISRTN registry (ISRCTN10417167). Participants will be sent a detailed participant information sheet and given at least 24 hours for consideration whether to partake in the study. The participant information sheet (Supplementary file 1) and the recruitment poster have been reviewed and approved by the ethics committee for complete and fair description of taking part. We will obtain written informed consent from all participants using an approved consent form (Supplementary file 2). Any protocol amendments will be submitted to the Oxford University Research and Ethics Committee and Clinical Trials and Research Governance teams for approval before being incorporated.

The findings from the study may be disseminated using academic media including peer-reviewed journal articles, national and international conference presentations, social media (including Twitter), electronic mail within the University of Oxford, and the internet including the departmental websites. The findings from the study will form part of a doctoral dissertation for MH. Authorship of journal articles related to this study will follow the ICMJE guidelines [15]. Due to the nature of the data being collected (video/image-based), it is impossible to completely anonymise the data source. This will be explicitly discussed during the informed consent. Participants will be identified only by a participant identification number on study documents and any electronic database. Documents that contain identifying data and/or information allowing linkage between participant identification number and personal data will be stored separately under strict access controls. The participants will retain the right to have their images deleted from storage at any time upon request.

Access to data

The data, stored in a proprietary format that is only readable by the review software, will be recorded onto an encrypted portable storage device that will be transferred by the researchers in secured storage to the University server. Except during transport, access to the data will be restricted to academic areas behind two limited access doors. All study documents will be stored securely and only accessible by study staff (Kadoorie Centre for Critical Care Research and Education, and the Institute of Biomedical Engineering/Oxford University Centre of Excellence for Medical Engineering) and authorised personnel from the University of Oxford for monitoring and/or audit of the study to ensure compliance with regulations. All video data will be stored securely using industry-standard encryption methods. The video data forms part of the research data and will be stored securely for 5 years after the release date of the last publication arising from the study.

Ancillary and post-trial care

If a participant in this study is ever considered to have suffered harm through their participation in the study, the University of Oxford as the sponsor has arrangements to provide compensation. Participants will be informed of potential route for escalation of any issues that arise from the study via the study team or via the Chair of the Medical Sciences Interdivisional Research Ethics Committee (MS IDREC) at the University of Oxford as appropriate.

For peer review only

Discussion

To the best of our knowledge, this is the first study aiming to create a skin surface perfusion map of the lower limbs and study changes to the map with increase and decrease in peripheral skin blood flow. This is one of a series of planned studies designed to answer how much information can be measured at accessible skin surface level using non-contact tools.

A limitation of this proposed study is that it is only including healthy volunteers in artificial controlled conditions with restricted movement. However, we feel that this is an important initial step as if no useful signal can be derived from the best possible environment with no movement artefacts, further efforts at looking for this absent signal in more realistic situations may not be fruitful. Creating optimum conditions by limiting movement will allow us to isolate signals that are present and potentially allow a better understanding of these signals of interest so that they can be isolated more effectively within noisy environment. Some concerns may be raised by the use of healthy population. The haemodynamic control requires a fine balance of our sympathetic and parasympathetic systems, and we know that this balance is not at baseline in the critically ill. However, the understanding of how the differing physiology in healthy and non-healthy populations translate to changes measured at skin surface will expand our understanding of critical illness and haemodynamic collapse.

Another key challenge is that by using specific receptor agonists, we bypass the physiological pathway by which the skin blood flow is reduced or increased. In healthy population used in the proposed study, we expect the vessel changes to cause subsequent changes in central cardiovascular balance. In critical illness, haemodynamic collapse and low blood pressure leads to peripheral vasoconstriction and reduced peripheral circulation. Creating the same peripheral conditions using phenylephrine infusion will lead to a rise in blood pressure. However, our methodology will allow us to see the pattern of reduction and increase in skin perfusion. This will provide invaluable information when similar changes are tracked on skin surface in patient populations.

For peer review only

References

1. van Genderen ME, Paauwe J, de Jonge J, *et al*. Clinical assessment of peripheral perfusion to predict postoperative complications after major abdominal surgery early: a prospective observational study in adults. *Crit Care* 2014;18(3):R114–R114.
2. Calzia E, Iványi Z, Radermacher P. Determinants of blood flow and organ perfusion. In: Functional hemodynamic monitoring. Springer 2005:19–32.
3. Van Genderen ME, Bartels SA, Lima A, *et al*. Peripheral perfusion index as an early predictor for central hypovolemia in awake healthy volunteers. *Anesth Analg* 2013;116(2):351–356.
4. Vazquez R, Vazquez Guillamet C, Adeel Rishi M, *et al*. Physical examination in the intensive care unit: opinions of physicians at three teaching hospitals. *Southwest J Pulm Crit Care* 2015;10(1):34–43.
5. Lima A, Jansen TC, van Bommel J, *et al*. The prognostic value of the subjective assessment of peripheral perfusion in critically ill patients. *Crit Care Med* 2009;37(3):934–938.
6. Lima A, Bakker J. Clinical monitoring of peripheral perfusion: there is more to learn. *Crit Care* 2014;18(1):113.
7. Harford M, Catherall J, Gerry S, *et al*. Availability and performance of image-based, non-contact methods of monitoring heart rate, blood pressure, respiratory rate, and oxygen saturation: A systematic review. *Physiol Meas* 2019;40(6):06TR01
8. Sun Y, Thakor N. Photoplethysmography Revisited: From Contact to Noncontact, From Point to Imaging. *IEEE Trans Biomed Eng* 2016;63(3):463–477.
9. Cooke WH, Moralez G, Barrera CR, *et al*. Digital infrared thermographic imaging for remote assessment of traumatic injury. *J Appl Physiol (1985)* 2011;111(6):1813–1818.
10. Richards LM, Kazmi SM, Davis JL, *et al*. Low-cost laser speckle contrast imaging of blood flow using a webcam. *Biomed Opt Express* 2013;4(10):2269–83.
11. Ash C, Dubec M, Donne K, *et al*. Effect of wavelength and beam width on penetration in light-tissue interaction using computational methods. *Lasers Med Sci* 2017;32(8):1909–1918.
12. Dünser MW, Dankl D, Petros S, *et al*. Clinical Examination Skills in the Adult Critically Ill Patient. Springer; 2018.
13. Taylor GI, Corlett RJ, Dhar SC, *et al*. The anatomical (angiosome) and clinical territories of cutaneous perforating arteries: development of the concept and designing safe flaps. *Plast Reconstr Surg* 2011;127(4):1447-59.
14. Chan A-W, Tetzlaff JM, Altman DG, *et al*. SPIRIT 2013 Statement: Defining standard protocol items for clinical trials. *Ann Intern Med* 2013;158:200-207.
15. Recommendations for the Conduct, Reporting, Editing, and Publication of Scholarly works in Journals. International Committee of Medical journal Editors. Available: http://icmje.org/recommendations [Accessed on 23 July 2019].

For peer review only

Supplementary files

- Supplementary file 1: Proposed Participant Information Sheet
- Supplementary file 2: Proposed Consent form

Authors' contributions

MH conceived the study with guidance from PW, DY, LT, MV and JJ. MH, CA, MV, JJ, EF, and SD will conduct screening and data collection. MH and AM planned the statistical analysis. Analysis will be performed by MH, MV, JJ, EF and SD. MH prepared the first draft of this manuscript. All authors critiqued and edited the manuscript for intellectual content.

Trial sponsor

University of Oxford, Oxford, United Kingdom

Funding statement

This work was supported by NIHR Oxford Biomedical Research Centre (BRC) under Technology & Digital Health theme. NIHR Oxford BRC advocates open access. MH, CA and PW are funded by NIHR Oxford BRC. The work of JJ was supported by the RCUK Digital Economy Programme, grant number EP/G036861/1 (Oxford Centre for Doctoral Training in Healthcare Innovation). JJ also acknowledges Fundacao para a Ciencia e Tecnologia, Portugal, doctoral grant SFRH/BD/85158/2012. MV was supported by the Oxford Centre of Excellence in Medical Engineering funded by the Wellcome Trust and EPSRC under grant number WT88877/Z/09/Z.

The funders have had no role in the study protocol design or the preparation of this manuscript, and will have no role in the collection, management, analysis and interpretation of the data, or the writing of the final report.

Competing interests statement

PW is Chief Medical Officer for Sensyne Health and holds share options in the company. His organisation has received research funding from Sensyne Health not associated with this work. LT is a non-executive Director of Sensyne Health and holds share options in the company. LT is a non-executive Director of Oxehealth and holds shares in the company.

Patient and Public Involvement

The aims of the study and its potential role in the design of future studies into haemodynamic monitoring within critical care was discussed with a critical care patient group based in Oxford University Hospitals NHS Foundation Trust.

For peer review only

Word count

3540

For peer review only

Critical Care Research Group (Kadoorie Centre)
Level 3, John Radcliffe Hospital, Oxford
Nuffield Department of Clinical Neurosciences
Tel: +44 (0)1865 231449
Email: mirae.harford@ndcn.ox.ac.uk

PARTICIPANT INFORMATION SHEET

Mapping Of Lower Limb skIn pErfusion (MOLLIE)

Ethics Approval Reference: R63796/RE001
Version 2.0
Date: May 2019

We'd like to invite you to take part in our research study. Before you decide, it is important that you understand why the research is being done and what it would involve for you. Please take time to read this information, and discuss it with others if you wish. If there is anything that is not clear, or if you *would like more information, please ask us.*

What is the purpose of the study?

We are trialling a new approach to monitoring how well a patient's heart and their cardiovascular system is functioning. Many established methods of looking at this use invasive monitors or semi-invasive monitors. Non-invasive monitors are less uncomfortable for patients but still require direct contact with the patients with risks of infection and skin irritation. The new method uses video cameras that can detect the way blood arrives at the skin surface, which is not visible to the naked eye. We believe that using this method we may be able to develop a way we can monitor patients without any invasive lines or contacts to their skin.

In order to explore this further, we are aiming to study the way skin is perfused in health, followed by simulating some of the changes that may occur when patients are less well. We are particularly interested in leg skin perfusion and that is the focus of this study. We are planning on using two methods to study this:

- Increasing and decreasing the amount of blood delivered to your skin surface by changing the diameter of blood vessels close to the skin surface. This will be done by using two drugs called phenylephrine and glyceryl trinitrate (GTN). Both of these drugs have been used safely in healthy volunteers and they are frequently used in hospitals. After a recovery period, we will roll a tight-fitting doughnut-shaped ring over the leg. It will feel like wearing tight-fitting socks or tights temporarily and should not be uncomfortable. The ring will act as a

tourniquet once placed around the thigh. After one minute this tourniquet will be released and the re-filling of these vessels will be monitored using the video camera.

Why have I been invited?

You have been invited because you are a healthy adult, aged between 18 and 65, do not have any known pre-existing heart problems, not on any regular medication (except oral contraception), and have expressed interest in taking part in the study.

You will not be able to take part if:

- there are any reasons why you cannot use the monitoring equipment (camera monitor or monitors which are attached to your body via sticky adhesive pads) due to skin disorders such as eczema, scleroderma or psoriasis
- you have had any degree of leg amputation
- you have had any previous surgery to the thigh/knee/lower leg (except knee arthroscopy)
- you are allergic to silver chloride ECG pads
- you have hyperthyroidism
- you are pregnant or breastfeeding
- you have neurological conditions affecting blood flow to the leg
- you have known heart conditions
- you suffer from severe headaches

Do I have to take part?

No. It is up to you to decide if you want to take part in the study. We will describe the study and go through this information sheet with you to answer any questions you may have.

If you agree to take part, we will ask you to sign a consent form and give you a copy for you to keep.

If you change your mind, you are free to withdraw from the study at any time, without having to provide a reason.

What will happen to me if I decide to take part?

The study will take part in the Cardiovascular Clinical Research Facility (John Radcliffe Hospital, Oxford) and will involve one single visit, unless we are unable to collect data needed on this visit or you ask us to discontinue the study and reschedule for another day. We expect one full session to take approximately 2.5 hours.

As we know that the video monitors are very sensitive to body hair, we will ask you to remove hair on your leg for the study. As shaving or waxing during the study visit may temporarily increase blood flow to the skin surface, we will ask you to do this at least 24 hours before the study visit. If you have hair removal equipment at home,

For peer review only

you can use your usual method. If not, we will discuss this with you before the study visit and agree on a plan. If required, we can supply body hair removal equipment (for shaving or waxing) that you can use.

Prior to starting, we will go through this information with you again and ask if you have any questions. Following that we will ask you some brief medical questions to ensure you are fit and well and examine your heart, lungs, and abdomen to make sure you are fit to take part in the study. Once we are happy that you can partake in the study, we will take some basic measurements from you. This will include your height, weight, and your skin colour from your arm according to a skin colour chart. This is so that the performance of the camera monitor can be compared to your skin tone. As we are recording from your legs, we will ask you to wear shorts so that the skin over your thigh is visible.

We will ask you to lie down on the bed and place a small drip on your arm. This is called a cannula and involves a small needle to ensure it sits inside your vein. The needle is removed after the drip is placed and a small plastic tube will then remain in your arm. The purpose of a cannula is to allow controlled infusion of the drugs to change the diameter of your blood vessels in a safe way. The cannulation will be completed by a trained doctor using sterile equipment. Once inserted, the cannula placement will be checked by flushing up to 10mls of normal saline (sterile fluid with small amount of salt to match the salt levels in the blood) and it will be secured using a specially designed plaster.

The cannula will then be connected to a flexible tube which will be connected to the drug infusion pump. At this point, the infusion will not be running.

The study will involve multiple methods of monitoring the heart and the circulation as described below:

- Video monitors: this is just like a normal video camera and will be mounted onto a trolley next to the examination bed where you will be lying. The camera will be set up after you are in the room and you will be warned when the recording is about to start. The camera is only recording your legs; your face and upper body will not be recorded. While the camera is recording we will ask you to maintain the same position as much as possible, as the monitor is very sensitive to movement. The camera will continue to record throughout the entire study visit and will be analysed at a later point.
- Thoracic bioimpedance monitor: This looks at the way your heart is pumping the blood around the body by measuring the total amount of water inside the chest cavity. The machine does this by passing a tiny amount of electrical signal between the leads which will be placed as sticky adhesives on either side of your neck and your lower chest. The amount of electrical signal passed is very small and you will not be aware of it. This is a continuous monitor and will continue to record throughout the study period.

For peer review only - http://bmjopen.bmj.com/site/about/guidelines.xhtml

- A non-invasive blood pressure monitor: This is a cuff that will be placed around your right arm which will intermittently inflate to measure your blood pressure.
- An oxygen saturation probe: This is an earlobe probe that will be placed on your left earlobe. The probe measures the amount of oxygen your blood is carrying using red and infrared light.
- Stowood Blackshadow monitor: This is a device which measures your breathing rate via two belts across your chest and a monitor placed just under your nostrils, and your heart rate and oxygen saturations using sticky adhesives on your chest and a finger probe.

Once all equipment is attached and we are ready to record, we will let you know when the study will start. For the first ten minutes, we will ask you to lie down as still as possible while we take some background measurements. We know from previous studies that talking during this period can cause fine movements in the body which reduces the quality of the images. Therefore, we will ask you to maintain silence during this period.

After ten minutes, we will administer one of the drugs. Each will last for 15 minutes and there will be a break of 20 minutes before the next drug is given. While the drug is being given, your blood pressure will be measured every minute. The drug infusion rate will slowly increase using our protocol, and we will stop increasing the rate once your blood pressure changes by 30% from baseline.

Once the drug infusions are complete and you have had a further break of 20 minutes, we will apply a tourniquet to one of your legs after passing a wrap balloon over your leg to empty out the blood vessels near the surface of your skin. After the tourniquet is applied for approximately 30 seconds, we will let the cuff down while taking a video of your leg.

This is the end of the study and after safe removal of the cannula, we will observe you for a further five minutes. We will offer you some refreshments during this observation period. If you are feeling well, you will be free to go.

What drugs will I be given?

- Phenylephrine is a drug which has two effects; it can increase your blood pressure and lower your heart rate. It raises blood pressure by reducing the diameter of small blood vessels of arms and legs). The infusion is frequently used in women having caesarean sections under spinal anaesthetic to keep the blood pressure stable, and has been used at higher doses than planned in this study in other healthy volunteer studies.
- Glyceryl trinitrate (GTN) is a drug which has the opposite effect to that of phenylephrine. It reduces blood pressure by dilating the blood vessels. It is used in clinical settings to reduce blood pressure and to reduce the work of

For peer review only

the heart when people have heart problems, and has been used in healthy volunteer studies at higher doses than planned in this study. Its main side effect is that in some people it can cause a headache. If this occurs, let us know and we will not further increase the dose.

Both of these drugs are short acting and will wash out of your system quickly once the infusions stop. It is very unlikely that you will have ongoing effects from the drug after leaving the study visit.

What should I consider?

You may participate in other research studies while you are taking part in this study.

Are there any possible disadvantages or risks from taking part?

The monitoring methods described are very safe and non-invasive. They have been trialled in healthy people and in patients without any problems.

The cannulation procedure and its presence may feel uncomfortable. We will not keep the cannula in for any longer than necessary. Once removed, the cannula may leave a small bruise but will heal quickly within 2-3 days.

There is a possibility that you may experience side effects from the drug infusions. The side effects occur because of the transient changes in blood pressure. More specifically, the most common side effects of the two drugs are headaches, flushing, and nausea. These effects should disappear once the drugs washout from your system. Approximately 95% of the drugs will be out of your system by the time you leave the study visit and the remainder (which is not enough to cause you any symptoms or harm) will leave your body over a few hours following the study visit. Other risks associated with very high or low blood pressure will be minimised by monitoring your blood pressure closely at one minute intervals and making small step increases in drug doses being given. We will not allow your blood pressure to deviate more than 30% from baseline which falls within ranges expected during activities such as exercise and sleep.

The tourniquet placed on the thigh will be inflated to high pressure to stop blood flow. This can be uncomfortable but will only last for 30 seconds before the cuff is deflated.

What are the possible benefits of taking part?

There is no direct benefit for your participation in this study. We hope that the results from this research will help us develop a new method of monitoring critically unwell patients in the hospital in a more comfortable way.

For peer review only

Will my taking part in the study be kept confidential?

Yes. All study data will be entered on a spreadsheet and you will only be identifiable by a unique study specific number and/or code in any database. The name and any other identifying detail about you will not be included in any study data electronic file.

The raw video data of your legs will be stored in a format that is only readable by the review software and will be recorded onto a storage device that will be physically secured at Kadoorie Centre (behind two separate locked doors with restricted access) and the Institute of Biomedical Engineering (behind a locked door with restricted access and 24 hour CCTV monitoring). Access to this storage device will be limited to our research team. Video data analysis will be completed by our collaborators at the Oxford University Centre of Excellence for Medical Engineering who have designed the equipment being used for the study. They are based in the Institute of Biomedical Engineering.

Will I be reimbursed for taking part?

Should you incur any expenses as a result of the study, we will reimburse you as is reasonable (e.g. travel expenses). Additionally, once you complete the study we will give you a £50 voucher.

What will happen to my data?

The information you provide as part of the study is the **research data**. Any research data from which you can be identified (e.g. your name, date of birth), is known as **personal data**. This includes more sensitive categories of personal data (**special category data**) such as your racial or ethnic origin or data concerning your health. This does not include data where the identity has been removed (anonymous data).

We will minimise our use of personal and sensitive data in the study as much as possible.

Consent forms (which include your name) will be stored in the Kadoorie Centre for Critical Care Research and Education within the John Radcliffe Hospital, behind two restricted access doors. All other research data will be stored in the Kadoorie Centre or in the Institute of Biomedical Engineering in the Department of Engineering Science, University of Oxford on a secure local server. Access to this server is restricted to the members of our study team only and the Institute of Biomedical Engineering is accessed via two restricted access doors. Research data will be anonymised. Personal/sensitive data will be stored in the Kadoorie Centre.

The researcher and the research team will have access to personal/sensitive/research data.

For peer review only - http://bmjopen.bmj.com/site/about/guidelines.xhtml

We would like your permission to use anonymised images or videos of your legs in research publications. While it is not possible to fully anonymise images of legs, we will not use images with any distinguishing features (e.g. distinctive scar) which may make you identifiable from the image.

All research data and records will be stored for a minimum of 5 years after publication or public release of the work of the research.

We would like your permission to use anonymised data in future studies, and to share data with other researchers (e.g. online database) both inside and outside the European Union. All personal information that could identify you will be removed or changed before information is shared with other researchers or results are made public.

The University of Oxford is the data controller with respect to your personal data and, as such, will determine how your personal data is used in the study. The University will process your personal data for the purpose of the research outlined above. Research is a task that we perform in the public interest.

For further information about your rights with respect to your personal data is available http://www.admin.ox.ac.uk/councilsec/compliance/gdpr/individualrights/.

What will happen if I don't want to carry on with the study?

Participation is voluntary you may change your mind at any stage. If you withdraw from the study, unless you state otherwise, any non-identifiable video data which have been collected whilst you have been in the study will be used for research as detailed in this participant information sheet. You are free to request that your video samples are destroyed at any time during or after the study.

What will happen to the results of this study?

It will not be possible to identify you from any report or publication placed in the public domain.

Once the study is complete including the analysis of results, the data will be used for publication in scientific journals and may be presented at conferences. It is also predicted that the current study will be used as basis for future similar studies.

Some of the research being undertaken will also contribute to the fulfilment of an educational requirement (i.e. a doctoral thesis).

For peer review only

For peer review only - http://bmjopen.bmj.com/site/about/guidelines.xhtml

What if we find something unexpected?

It is important to note that the examination and monitoring in the study are carried out for research purposes rather than for diagnosis. Therefore, participating in the study is not a substitute for a doctor's appointment. Occasionally, however, a possible abnormality may be detected. In this case, we will inform you of the findings and inform the lead investigator (Professor Peter Watkinson). If the lead investigator felt that the abnormality was medically important, you will be recommended to see your general practitioner. All information about you is kept strictly confidential.

There is no need to inform your General Practitioner/family doctor of your participation in the study unless you would like to. In the unlikely event that any findings need urgent treatment or investigation, we will inform you of this and may speak to your GP with your permission.

What if there is a problem?

If a participant in research is ever considered to have suffered harm through their participation, the University has arrangements in place to provide for compensation. If you have a concern about any aspect of this study, please speak to the relevant researcher (01865 231449) or their supervisor (01865 572609), who will do their best to answer your query. The researcher should acknowledge your concern within 10 working days and give you an indication of how they intend to deal with it. If you remain unhappy or wish to make a formal complaint, please contact the Chair of the Medical Sciences Interdivisional Research Ethics Committee (MS IDREC) at the University of Oxford who will seek to resolve the matter in a reasonably expeditious manner: Email: ethics@medsci.ox.ac.uk; Address: Research Services, University of Oxford, Wellington Square, Oxford OX1 2JD.

Who is organising and funding the study?

This study is being organised by a group of researchers specialising in critical care research at the Nuffield Department of Clinical Neuroscience, based at Kadoorie Centre in John Radcliffe Hospital.

The study is funded by the Oxford Biomedical Research Centre, which is a part of National Institute for Health Research (NIHR).

Who has reviewed the study?

This study has been reviewed by, and received ethics clearance through, the University of Oxford Central University Research Ethics Committee (Reference number: R63796/RE001).

Participation in future research:

We will not keep your contact details to contact you regarding future research. If you would like to know more about participating in future research projects, please contact Dr Mirae Harford.

For peer review only

For peer review only - http://bmjopen.bmj.com/site/about/guidelines.xhtml

Further information and contact details:

Please contact Dr Mirae Harford by email: mirae.harford@ndcn.ox.ac.uk.

Thank you for reading this information and for considering taking part.

For peer review only

Information sheet<br>*Skin perfusion mapping in volunteers*<br>*Mirae Harford*	Version/Date: 3.0/1.9.19<br>Ethics Ref: R63796/RE001

Critical Care Research Group
Nuffield Department of Clinical Neurosciences
University of Oxford

Lead investigator: Professor Peter Watkinson
Peter.watkinson@ndcn.ox.ac.uk
Primary researcher: Mirae Harford
Oxford telephone number: 01865 231449
Oxford e-mail: mirae.harford@ndcn.ox.ac.uk

PARTICIPANT CONSENT FORM

CUREC Approval Reference: R63796/RE001

Mapping Of Lower Limb skIn pErfusion (MOLLIE)

Purpose of Study: To investigate leg skin perfusion at rest and changes created to the perfusion pattern in response to phenylephrine and glyceryl trinitrate (GTN) infusions.

		Please initial each box
1	I confirm that I have read and understand the information sheet version 2.0 dated May 2019 for the above study. I have had the opportunity to consider the information carefully, ask questions and have had these questions answered satisfactorily.	
2	I understand that my participation is voluntary and that I am free to withdraw at any time, without giving any reason, and without any adverse consequences or academic penalty.	
3	I have been advised about the potential risks associated with taking part in this research and have taken these into consideration before consenting to participate. I understand that the study visit involves video equipment and the recordings will be saved for subsequent analysis.	
4	I have been advised as to what I need to do for this research (especially with regard to the phenylephrine and GTN administration) and I agree to follow the instructions given to me. I understand that taking part will involve having a drip and application of a tourniquet on one leg which will stay inflated for one minute.	
5	To the best of my knowledge, I do not meet any of the exclusion criteria outlined in the information sheet for this research. If this changes at a later date during study participation, I agree to notify the researchers immediately.	
6	I understand that data collected during the study may be looked at by designated individuals from the University of Oxford. I give permission for these individuals to access my data.	
8	I understand who will have access to personal data provided, how the data will be stored and what will happen to the data at the end of the project.	
9	I agree for data collected in this study to be shared with other researchers, including those working outside of the EU, to be used in other research studies. I understand that any data shared will be fully anonymised so that I cannot be identified.	

For peer review only - http://bmjopen.bmj.com/site/about/guidelines.xhtml

10	I understand how this research will be written up and published. I agree to the use of anonymised data (including videos) in research publications.	☐
11	I understand that this project has been reviewed by, and received ethics clearance through, the University of Oxford Central University Research Ethics Committee.	☐
12	I understand how to raise a concern or make a complaint.	☐
13	I agree to take part in the study	☐

Name of Participant	dd / mm / yyyy<br>Date	Signature
Name of person taking consent	dd / mm / yyyy<br>Date	Signature

For peer review only

STANDARD PROTOCOL ITEMS: RECOMMENDATIONS FOR INTERVENTIONAL TRIALS

SPIRIT 2013 Checklist for Mapping Of Lower Limb skin pErfusion (MOLLIE): Recommended items to address in a clinical trial protocol and related documents*

Section/item	Item No	Description	Addressed on page number
**Administrative information**			
Title	1	Descriptive title identifying the study design, population, interventions, and, if applicable, trial acronym	1
Trial registration	2a	Trial identifier and registry name. If not yet registered, name of intended registry	2, 10
	2b	All items from the World Health Organization Trial Registration Data Set	ISRCTN.com
Protocol version	3	Date and version identifier	1
Funding	4	Sources and types of financial, material, and other support	13
Roles and responsibilities	5a	Names, affiliations, and roles of protocol contributors	1, 13
	5b	Name and contact information for the trial sponsor	13
	5c	Role of study sponsor and funders, if any, in study design; collection, management, analysis, and interpretation of data; writing of the report; and the decision to submit the report for publication, including whether they will have ultimate authority over any of these activities	13
	5d	Composition, roles, and responsibilities of the coordinating centre, steering committee, endpoint adjudication committee, data management team, and other individuals or groups overseeing the trial, if applicable (see Item 21a for data monitoring committee)	N/A

For peer review only

**Introduction**			
Background and rationale	6a	Description of research question and justification for undertaking the trial, including summary of relevant studies (published and unpublished) examining benefits and harms for each intervention	4 – 5
	6b	Explanation for choice of comparators	4 – 5
Objectives	7	Specific objectives or hypotheses	5
Trial design	8	Description of trial design including type of trial (eg, parallel group, crossover, factorial, single group), allocation ratio, and framework (eg, superiority, equivalence, noninferiority, exploratory)	5
**Methods: Participants, interventions, and outcomes**			
Study setting	9	Description of study settings (eg, community clinic, academic hospital) and list of countries where data will be collected. Reference to where list of study sites can be obtained	6
Eligibility criteria	10	Inclusion and exclusion criteria for participants. If applicable, eligibility criteria for study centres and individuals who will perform the interventions (eg, surgeons, psychotherapists)	6
Interventions	11a	Interventions for each group with sufficient detail to allow replication, including how and when they will be administered	6 – 7
	11b	Criteria for discontinuing or modifying allocated interventions for a given trial participant (eg, drug dose change in response to harms, participant request, or improving/worsening disease)	7
	11c	Strategies to improve adherence to intervention protocols, and any procedures for monitoring adherence (eg, drug tablet return, laboratory tests)	N/A
	11d	Relevant concomitant care and interventions that are permitted or prohibited during the trial	N/A
Outcomes	12	Primary, secondary, and other outcomes, including the specific measurement variable (eg, systolic blood pressure), analysis metric (eg, change from baseline, final value, time to event), method of aggregation (eg, median, proportion), and time point for each outcome. Explanation of the clinical relevance of chosen efficacy and harm outcomes is strongly recommended	8
Participant timeline	13	Time schedule of enrolment, interventions (including any run-ins and washouts), assessments, and visits for participants. A schematic diagram is highly recommended (see Figure)	5 – 7

For peer review only - http://bmjopen.bmj.com/site/about/guidelines.xhtml

Sample size	14	Estimated number of participants needed to achieve study objectives and how it was determined, including clinical and statistical assumptions supporting any sample size calculations	6
Recruitment	15	Strategies for achieving adequate participant enrolment to reach target sample size	6

Methods: Assignment of interventions (for controlled trials)

Allocation:

Sequence generation	16a	Method of generating the allocation sequence (eg, computer-generated random numbers), and list of any factors for stratification. To reduce predictability of a random sequence, details of any planned restriction (eg, blocking) should be provided in a separate document that is unavailable to those who enrol participants or assign interventions	N/A
Allocation concealment mechanism	16b	Mechanism of implementing the allocation sequence (eg, central telephone; sequentially numbered, opaque, sealed envelopes), describing any steps to conceal the sequence until interventions are assigned	N/A
Implementation	16c	Who will generate the allocation sequence, who will enrol participants, and who will assign participants to interventions	N/A
Blinding (masking)	17a	Who will be blinded after assignment to interventions (eg, trial participants, care providers, outcome assessors, data analysts), and how	N/A
	17b	If blinded, circumstances under which unblinding is permissible, and procedure for revealing a participant's allocated intervention during the trial	N/A

Methods: Data collection, management, and analysis

Data collection methods	18a	Plans for assessment and collection of outcome, baseline, and other trial data, including any related processes to promote data quality (eg, duplicate measurements, training of assessors) and a description of study instruments (eg, questionnaires, laboratory tests) along with their reliability and validity, if known. Reference to where data collection forms can be found, if not in the protocol	7 – 8
	18b	Plans to promote participant retention and complete follow-up, including list of any outcome data to be collected for participants who discontinue or deviate from intervention protocols	7 – 8

Data management	19	Plans for data entry, coding, security, and storage, including any related processes to promote data quality (eg, double data entry; range checks for data values). Reference to where details of data management procedures can be found, if not in the protocol	7 – 9
Statistical methods	20a	Statistical methods for analysing primary and secondary outcomes. Reference to where other details of the statistical analysis plan can be found, if not in the protocol	8 – 9
	20b	Methods for any additional analyses (eg, subgroup and adjusted analyses)	8 – 9
	20c	Definition of analysis population relating to protocol non-adherence (eg, as randomised analysis), and any statistical methods to handle missing data (eg, multiple imputation)	7 – 8
**Methods: Monitoring**			
Data monitoring	21a	Composition of data monitoring committee (DMC); summary of its role and reporting structure; statement of whether it is independent from the sponsor and competing interests; and reference to where further details about its charter can be found, if not in the protocol. Alternatively, an explanation of why a DMC is not needed	10
	21b	Description of any interim analyses and stopping guidelines, including who will have access to these interim results and make the final decision to terminate the trial	N/A
Harms	22	Plans for collecting, assessing, reporting, and managing solicited and spontaneously reported adverse events and other unintended effects of trial interventions or trial conduct	8
Auditing	23	Frequency and procedures for auditing trial conduct, if any, and whether the process will be independent from investigators and the sponsor	10
**Ethics and dissemination**			
Research ethics approval	24	Plans for seeking research ethics committee/institutional review board (REC/IRB) approval	10
Protocol amendments	25	Plans for communicating important protocol modifications (eg, changes to eligibility criteria, outcomes, analyses) to relevant parties (eg, investigators, REC/IRBs, trial participants, trial registries, journals, regulators)	10

For peer review only - http://bmjopen.bmj.com/site/about/guidelines.xhtml

Consent or assent	26a	Who will obtain informed consent or assent from potential trial participants or authorised surrogates, and how (see Item 32)	10
	26b	Additional consent provisions for collection and use of participant data and biological specimens in ancillary studies, if applicable	N/A
Confidentiality	27	How personal information about potential and enrolled participants will be collected, shared, and maintained in order to protect confidentiality before, during, and after the trial	10
Declaration of interests	28	Financial and other competing interests for principal investigators for the overall trial and each study site	13
Access to data	29	Statement of who will have access to the final trial dataset, and disclosure of contractual agreements that limit such access for investigators	10
Ancillary and post-trial care	30	Provisions, if any, for ancillary and post-trial care, and for compensation to those who suffer harm from trial participation	10
Dissemination policy	31a	Plans for investigators and sponsor to communicate trial results to participants, healthcare professionals, the public, and other relevant groups (eg, via publication, reporting in results databases, or other data sharing arrangements), including any publication restrictions	10
	31b	Authorship eligibility guidelines and any intended use of professional writers	10 (ICMJE)
	31c	Plans, if any, for granting public access to the full protocol, participant-level dataset, and statistical code	N/A
**Appendices**			
Informed consent materials	32	Model consent form and other related documentation given to participants and authorised surrogates	Appendix
Biological specimens	33	Plans for collection, laboratory evaluation, and storage of biological specimens for genetic or molecular analysis in the current trial and for future use in ancillary studies, if applicable	N/A

*It is strongly recommended that this checklist be read in conjunction with the SPIRIT 2013 Explanation & Elaboration for important clarification on the items. Amendments to the protocol should be tracked and dated. The SPIRIT checklist is copyrighted by the SPIRIT Group under the Creative Commons "Attribution-NonCommercial-NoDerivs 3.0 Unported" license.

For peer review only - http://bmjopen.bmj.com/site/about/guidelines.xhtml
